# Association of Sperm-Associated Antigen 5 and Treatment Response in Patients With Estrogen Receptor–Positive Breast Cancer

**DOI:** 10.1001/jamanetworkopen.2020.9486

**Published:** 2020-07-07

**Authors:** Tarek M. A. Abdel-Fatah, Graham R. Ball, Pulari U. Thangavelu, Lynne E. Reid, Amy E. McCart Reed, Jodi M. Saunus, Pascal H. G. Duijf, Peter T. Simpson, Sunil R. Lakhani, Lorinc Pongor, Balázs Győrffy, Paul M. Moseley, Andrew R. Green, Alan G. Pockley, Carlos Caldas, Ian O. Ellis, Stephen Y. T. Chan

**Affiliations:** 1 Department of Clinical Oncology, Nottingham University Hospitals NHS Trust, Nottingham, United Kingdom; 2 Department of Pathology, National Liver Institute, Menoufyia University, Al Minufya, Egypt; 3 John van Geest Cancer Research Centre, Nottingham Trent University School of Science and Technology, Nottingham United Kingdom; 4 Diamantina Institute, Translational Research Institute, The University of Queensland, Brisbane, Australia; 5 UQ Centre for Clinical Research, Faculty of Research, The University of Queensland, Herston, Australia; 6 Pathology Queensland, The Royal Brisbane and Women’s Hospital, Herston, Australia; 7 Lendület Cancer Biomarker Research Group, Second Department of Pediatrics, Semmelweis University, Budapest, Hungary; 8 Nottingham Breast Cancer Research Center, Division of Cancer and Stem Cells, School of Medicine, University of Nottingham Biodiscovery Institute, University Park, Nottingham, United Kingdom; 9 Department of Oncology and Cancer Research, UK Cambridge Institute, Li Ka Shing Centre, University of Cambridge, Cambridge, United Kingdom

## Abstract

**Importance:**

There is no proven test that can guide the optimal treatment, either endocrine therapy or chemotherapy, for estrogen receptor–positive breast cancer.

**Objective:**

To investigate the associations of sperm-associated antigen 5 (*SPAG5*) transcript and SPAG5 protein expressions with treatment response in systemic therapy for estrogen receptor–positive breast cancer.

**Design, Settings, and Participants:**

This retrospective cohort study included patients with estrogen receptor–positive breast cancer who received 5 years of adjuvant endocrine therapy with or without neoadjuvant anthracycline-based combination chemotherapy (NACT) derived from 11 cohorts from December 1, 1986, to November 28, 2019. The associations of *SPAG5* transcript and SPAG5 protein expression with pathological complete response to NACT were evaluated, as was the association of *SPAG5* mRNA expression with response to neoadjuvant endocrine therapy. The associations of distal relapse–free survival with *SPAG5* transcript or SPAG5 protein expressions were analyzed. Data were analyzed from September 9, 2015, to November 28, 2019.

**Main Outcomes and Measures:**

The primary outcomes were breast cancer–specific survival, distal relapse–free survival, pathological complete response, and clinical response. Outcomes were examined using Kaplan-Meier, multivariable logistic, and Cox regression models.

**Results:**

This study included 12 720 women aged 24 to 78 years (mean [SD] age, 58.46 [12.45] years) with estrogen receptor–positive breast cancer, including 1073 women with *SPAG5* transcript expression and 361 women with SPAG5 protein expression of locally advanced disease stage IIA through IIIC. Women with *SPAG5* transcript and SPAG5 protein expressions achieved higher pathological complete response compared with those without *SPAG5* transcript or SPAG5 protein expressions (transcript: odds ratio, 2.45 [95% CI, 1.71-3.51]; *P* < .001; protein: odds ratio, 7.32 [95% CI, 3.33-16.22]; *P* < .001). Adding adjuvant anthracycline chemotherapy to adjuvant endocrine therapy for *SPAG5* mRNA expression in estrogen receptor–positive breast cancer was associated with prolonged 5-year distal relapse–free survival in patients without lymph node involvement (hazard ratio, 0.34 [95% CI, 0.14-0.87]; *P* = .03) and patients with lymph node involvement (hazard ratio, 0.35 [95% CI, 0.18-0.68]; *P* = .002) compared with receiving 5-year endocrine therapy alone. Mean (SD) *SPAG5* transcript was found to be downregulated after 2 weeks of neoadjuvant endocrine therapy compared with pretreatment levels in 68 of 92 patients (74%) (0.23 [0.18] vs 0.34 [0.24]; *P* < .001).

**Conclusions and Relevance:**

These findings suggest that *SPAG5* transcript and SPAG5 protein expressions could be used to guide the optimal therapies for estrogen receptor–positive breast cancer. Retrospective and prospective clinical trials are warranted.

## Key Points

**Question:**

Are sperm-associated antigen 5 (*SPAG5*) transcript or protein expressions associated with treatment response in patients with estrogen receptor–positive breast cancer?

**Findings:**

In this cohort study including 12 720 patients with estrogen receptor–positive breast cancer, *SPAG5* transcript and SPAG5 protein overexpressions were associated with worse outcomes in patients who received endocrine therapy alone. Overexpressions of *SPAG5* transcript or SPAG5 protein were associated with resistance to endocrine therapy but sensitivity to anthracycline-based combination chemotherapy, and downregulation of *SPAG5* during the course of preoperative systemic therapies was associated with clinical benefit.

**Meaning:**

These findings suggest that *SPAG5* transcript or SPAG5 protein expression could be used as a clinical tool for selecting and monitoring response to neoadjuvant therapies and guide adjuvant therapy in estrogen receptor–positive breast cancer.

## Teaser

This cohort study examines the association of sperm-associated antigen 5 (*SPAG5*) transcript and protein expression with treatment response in patients with estrogen receptor–positive breast cancer.

## Introduction

Among 1.38 million newly diagnosed breast cancer cases each year, 65% to 70% of them are estrogen receptor positive.^1^ Although single-agent endocrine therapy has significantly extended survival for patients with estrogen receptor–positive breast cancer, resistance to endocrine therapy is common, reported in up to 50% of patients.^2^ To extend treatment benefit and delay the development of endocrine therapy resistance, a combination of endocrine therapy with cytotoxic chemotherapy has been proven to be effective in up to 30% of estrogen receptor–positive breast cancers.^3,4,5,6^

Currently, there is no proven test that can accurately predict response to endocrine therapy or chemotherapy. The current practice is largely based on assessment of the recurrence risk and overall survival (OS), using traditional clinicopathological prognostic factors (eg, lymph node status) and multigene tests (eg, Oncotype DX [Genomic Health], MammaPrint [Agendia], and Prosigna [Nanostring Technologies]).^7^ However, these tests are used to assess outcomes and do not predict if a patient will respond to endocrine therapy or chemotherapy; therefore, it can be difficult for clinicians and patients to determine the risk/benefit ratio associated with endocrine therapy or chemotherapy or to select the effective treatment for individual patients. Therefore, there is a need for an improved method of determining if an individual is likely to respond to chemotherapy or endocrine therapy.

Previously, we reported that sperm-associated antigen 5 (*SPAG5*; OMIM 615562) is a novel oncogene in estrogen receptor–positive luminal-B subtype breast cancer.^8,9^ The aim of this study was to analyze the association of *SPAG5* gene and SPAG5 protein expression in estrogen receptor–positive breast cancer with treatment response, which may enable better management of estrogen receptor–positive breast cancer.

## Methods

### Study Design and Cohorts

**Figure 1.**
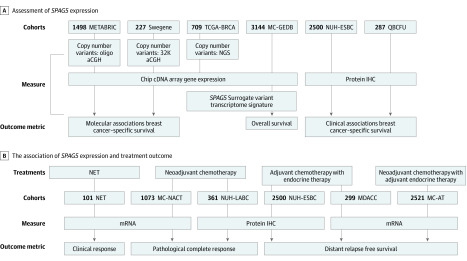
Cohort Flow Diagram aCGH indicates array comparative genomic hybridization; AT, adjuvant therapy; IHC, immunohistochemistry; MC-GEDB, multicenter Gene Expression databases; MC-NACT, multicenter neoadjuvant anthracycline combination chemotherapy; MDACC, MD Anderson Cancer Center; METABRIC indicates Molecular Taxonomy of Breast Cancer International Consortium; NACT, neoadjuvant anthracycline-based combination chemotherapy; NET, neoadjuvant endocrine therapy; NGS, next generation sequencing; NUH-ESBC, Nottingham University Hospital early stage breast cancer; NUH-LABC, Nottingham University Hospital locally advanced breast cancer; QBCFU, Queensland breast cancer follow-up; *SPAG5*, sperm-associated antigen 5; and TCGA-BRCA, The Cancer Genome Atlas-Breast Cancer project.

This study was approved by the institutional review board, independent ethics committee, or hospital research and innovations department at all participating sites. Oral and written consent was obtained from participants prior to the investigation. The participants did not receive financial compensation. This study followed the Strengthening the Reporting of Observational Studies in Epidemiology (STROBE) reporting guideline for cohort studies. The study design and patient cohorts are summarized in Figure 1.

### Patient Cohorts

The study was conducted in 11 cohorts of women with estrogen receptor–positive breast cancer from December 1, 1986, to November 28, 2019 (eAppendix in the Supplement): the Molecular Taxonomy of Breast Cancer International Consortium (METABRIC) cohort^10,11^; The Cancer Genome Atlas-Breast Cancer project (TCGA-BRCA) cohort^12^; the Swegene cohort^13^; the multicenter Gene Expression databases (MC-GEDBs) cohort, which was derived from published microarray data sets as previously described^14^; the Nottingham University Hospital early stage breast cancers (NUH-ESBC) cohort; the estrogen receptor–positive Queensland breast cancer follow-up (QBCFU) cohort; the neoadjuvant endocrine therapy (NET) cohort, which included patients who received aromatase inhibitors and was derived from 3 published microarray data sets (ie, GSE59515, GSE55374 and GSE20181) as previously described^15^; the multicenter neoadjuvant anthracycline combination chemotherapy (MC-NACT) cohort, which was derived from 11 databases; the NUH locally advanced breast cancer (NUH-LABC) cohort; the MD Anderson Cancer Center (MDACC) cohort,^16^ which included patients with recently diagnosed estrogen receptor–positive breast cancer who did not have *HER2* (OMIM 164870) gene expression and who were treated with NACT with taxane followed by 5-year adjuvant endocrine therapy^16^; and the multicenter adjuvant therapy (MC-AT) cohort, which was retrieved from 17 multicenter databases of patients with estrogen receptor–positive early stage breast cancer who had received systemic adjuvant therapy.

### Measurement and Procedures

#### *SPAG5* Copy Number Variants

Copy number variants at the *SPAG5* locus on chromosome 17q11.2 were retrieved from the METABRIC,^10,11^ TCGA-BRCA,^12^ and Swegene consortium^13^ cohorts. For the Swegene cohort, bacterial artificial chromosome microarrays were produced by the SCIBLU Genomics Centre, Lund University, Lund, Sweden.^13^ For the METABRIC and TCGA-BRCA cohorts, breast cancers were assayed using Affymetrix 6.0 SNP arrays, and the whole exome sequencing data were obtained for the TCGA-BRCA cohort.^12^ The aligned TCGA-BRCA data sets were downloaded using the CGHub download client software GeneTorrent version 3.8.5 as previously described.^14^

#### *SPAG5* Surrogate Transcriptomic Signature

The *SPAG5* surrogate transcriptomic signature, which corresponded to each type of *SPAG5* variant and included upregulated and downregulated genes, was identified using *SPAG5* genotype. Data on RNA sequence were derived from next generation sequencing in the TCGA-BRCA cohort, as previously described.^14^

#### *SPAG5* Gene Expression

Expression of *SPAG5* in mRNA was determined in publicly available gene expression data, including the METABRIC, TCGA-BRCA, Swegene, NET, MC-NACT, MDACC, and MC-AT cohorts. The gene expression raw data were downloaded using the raw data format as described previously.^8,14^

#### SPAG5 Protein Expression

Immunohistochemistry (IHC) of SPAG5 protein expression and other breast cancer biomarkers were investigated in the NUH-LABC, NUH-ESBR, and QBCFU cohorts (eTable 1 in the Supplement).^5^ SPAG5 protein expression was also evaluated in post-NACT surgical specimens from patients who had not achieved pathological complete response (CR) in the NUH-LABC cohort. Immunohistochemistry was performed using an anti-SPAG5 antibody (HPA022479; Sigma) at a dilution of 1:50.

### Outcome Measurements

Molecular characteristic associations of *SPAG5* expression (ie, copy number variants and mRNA*)* were determined in the METABRIC, TCGA-BRCA, and Swegene cohorts. The clinicopathological associations of SPAG5 protein expression were evaluated in the NUH-ESBC and QBCFU cohorts.

#### Survival

We defined OS as the number of months from diagnosis to the occurrence of death. Survival was censored if the patient was still alive or lost to follow-up by November 28, 2019. The association of *SPAG5* variant transcriptomic signature with OS was analyzed in the MC-GEDBs cohort, as previously described.^14^

Breast cancer–specific survival (BCSS) was defined as the number of months from diagnosis to the occurrence of death caused by breast cancer. The association of the SPAG5 protein expression with BCSS was analyzed in the METABRIC, Swegene, NUH-ESBC, and QBCFU cohorts.

Distant relapse free survival (DRFS) was defined as the number of months from diagnosis to distant metastases relapse. The association of prechemotherapy *SPAG5* mRNA expression with DRFS was evaluated in the MDACC cohort. The association of DRFS with *SPAG5* mRNA expression was tested in the MC-AT cohort, and the association of SPAG5 protein expression was tested in the NUH-ESBC cohort. The associations of DRFS with SPAG5 protein expressions in pre- and post-NACT tissue samples were analyzed in NUH-LABC cohort.

#### Treatment Response

Serial dynamic clinical response to neoadjuvant endocrine therapy was determined in the NET cohort by measuring tumor volumes during a 3-month treatment period and verified by mammographic measurements. Nonresponse was defined by an increase in tumor volume or a partial reduction that never exceeded 50%.^15^ The associations of *SPAG5* mRNA expression before (after 2 weeks) and during (after 3 months) treatment with clinical response were evaluated.

Pathological CR rate was defined as absence of any neoplastic cells in the primary breast site and lymph nodes after receiving NACT. The association of *SPAG5* mRNA expression with pathological CR was evaluated in the MC-NACT cohort. The associations of SPAG5 protein expression in the pre-NACT diagnostic core biopsies with pathological CR after receiving NACT were investigated in the NUH-LABC cohort.

### Statistical Analysis

Statistical analyses were performed using Statistica (Stat Soft ) and SPSS version 17 (IBM). Where appropriate, Pearson χ^2^, *t* test, and analysis of variance tests were used. Expression of SPAG5 protein before and after chemotherapy was calculated and compared using McNemar test. Cumulative survival probabilities, 10-year OS, and 5-year DRFS were estimated using the univariate Cox proportional hazards models and the Kaplan-Meier plot method where appropriate, and differences between survival rates were tested for significance using the log-rank test. Multivariable analysis for survival was performed using the Cox proportional hazard model. The proportional hazards assumption was tested using standard log-log plots. Hazard ratios (HRs) and 95% CIs were estimated for each variable. All tests were 2-sided, and *P* < .05 was considered statistically significant. The associations of *SPAG5* transcript and SPAG5 protein expressions with chemotherapy were tested using Cox proportional hazard model. For multiple comparisons, *P* values were adjusted according to Benjamini-Hochberg method. Data were analyzed from September 9, 2015, to November 28, 2019.

## Results

This study included 12 720 women aged 24 to 78 years (mean [SD] age, 58.46 [12.45] years) with estrogen receptor–positive breast cancer derived from 11 cohorts. The METABRIC cohort^10,11^ included 1498 patients with median (interquartile range [IQR]) follow-up time of 9.1 (5.2-12.9) years (eTable 2 in the Supplement). The TCGA-BRCA cohort^12^ included 381 patients with a median (IQR) follow-up time of 1.9 (1.7-3.6) years (eTable 3 in the Supplement). The Swegene cohort^13^ included 227 patients with a median (IQR) follow-up time of 8.1 (5.0-14.0) years (eTable 4 in the Supplement). The MC-GEDBs cohort^14^ included 3144 patients. There were 2500 patients in the NUH-ESBC cohort, including 1175 patients (47%) who were adjuvant therapy–naive, 1050 patients (42%) who received 5-year adjuvant endocrine therapy alone, and 275 patients (11%) who received adjuvant endocrine therapy and chemotherapy. Among 2105 patients in the NUH-ESBC cohort with *HER2* gene expression (7%), none received trastuzumab (eTable 5 in the Supplement). Median (IQR) follow-up time in the NUH-ESBC cohort was 13.4 (10.3-16.4) years. The QBCFU cohort included 287 patients. There were 101 patients in the NET cohort^15^ (eTable 6 in the Supplement). The MC-NACT cohort included 1073 patients, among whom 754 (70%) received NACT with taxane, 268 (25%) received NACT alone, and 51 (5%) received NACT with taxane and trastuzumab (eTable 7 and eTable 8 in the Supplement). The NUH-LABC cohort included 361 patients with a median (IQR) follow-up time of 5.1 (3.4-7.0) years, among whom 207 (58%) received NACT with taxane, 104 (29%) received NACT alone, and 45 (13%) received NACT with taxane and trastuzumab. Additionally, 25 patients (7%) did not receive adjuvant therapy, 180 patients (51%) received adjuvant endocrine therapy alone, and 151 patients (42%) received adjuvant endocrine therapy with chemotherapy. A total of 103 patients with *HER2* expression (29%) received adjuvant trastuzumab (eTable 9 in the Supplement). The MDACC cohort included 299 patients, and median (IQR) follow-up time was 9.0 (5.7-10.5) years (eTable 10 in the Supplement). The MC-AT cohort included 2521 patients with median (IQR) follow-up time of 5.4 (3.0-8.7) years (eTable 11 and eTable 12 in the Supplement). Among patients in the MC-AT cohort, 1408 patients (75%) received adjuvant endocrine therapy alone, including 1376 patients (75%) who received tamoxifen and 32 patients (2%) who received aromatase inhibitors, whereas 394 patients (28%) received chemotherapy in addition to adjuvant endocrine therapy; 253 patients (18%) received anthracycline with taxane, 85 patients (6%) received anthracycline alone, and 56 patients (4%) received cyclophosphamide, methotrexate, and fluorouracil. Among 282 patients with *HER2* expression (20%), only 11 patients (4%) received adjuvant trastuzumab.

Gain-amplification of *SPAG5* gene locus at chromosome 17q11.2 was more common in PAM50 luminal-B vs luminal-A (TCGA-BRCA: 72 of 230 patients [31%] vs 65 of 479 patients [14%]; *P* < .001; METABRIC: 87 of 488 patients [18%] vs 45 of 718 patients [6%]; *P* < .001; Swegene: 10 of 64 patients [16%] vs 1 of 89 patients [1%]; *P* < .001). Expression of *SPAG5* in mRNA was associated with luminal-B (determined via PAM50 and 4-IHC), *TP53* (OMIM 191170) variation, *HER2* expression, *BRCA2* (OMIM 600185) variation, luminal-complex genomic pattern, 17q12 genomic patterns, and high genomic instability integrative clusters (IntClust 1, 2 5, 6, 9 and 10) in the METABRIC and Swegene cohorts (eTable 13 and eTable 14 in the Supplement). In contrast, no *SPAG5* expression was associated with luminal-A, paucity of genomic changes, luminal-simplex genomic pattern (1q positive and 16q negative), low genomic instability, and IntClust 3, 4, and 8 (METABRIC and Swegene cohorts) (eTable 13 and eTable 14 in the Supplement). Expression of SPAG5 in protein was associated with luminal-B (4-IHC), *HER2* expression, and *TP53* variation (eTable 15 in the Supplement).

Expressions of *SPAG5* in copy number variant gain-amplification and mRNA and SPAG5 protein expression were associated with shorter BCSS compared with *SPAG5* copy number variants loss-neutral (METABRIC: HR, 1.55 [95% CI, 1.18-2.04]; *P* < .001; Swegene: HR, 2.27 [95% CI, 1.14-4.45]; *P* = .03), no *SPAG5* expression in mRNA (METABRIC: HR, 1.65 [95% CI, 1.31-2.09]; *P* < .001; Swegene: HR, 3.20 [95% CI, 2.25-5.70]; *P* < .001), and no SPAG5 expression in protein (NUH-ESBC: HR, 1.90 [95% CI, 1.51-2.47]; *P* < .001; QBCFU: HR, 2.57 [95% CI, 1.49-4.42]; *P* = .02) (eFigure 1 in the Supplement).

*SPAG5* transcript expression was associated with shorter BCSS compared with no *SPAG5* transcript expression in disease without lymph node involvement (METABRIC: HR, 2.07 [95% CI, 1.39-3.08]; *P* < .001) or with lymph node involvement (METABRIC: HR, 1.40 [95% CI, 1.05-1.87]; *P* = .02). Similarly, SPAG5 protein expression was associated with shorter BCSS compared with no SPAG5 protein expression in disease without lymph node involvement (NUH-ESBC: HR, 2.21 [95% CI, 1.52-3.21]; *P* < .001) or with lymph node involvement (NUH-ESBC: HR, 1.70 [95% CI, 1.25-2.39]; *P* < .001) (eFigure 2 in the Supplement).

In the MC-GEDs cohort, high *SPAG5* amplification signature was significantly associated with shorter OS compared with low *SPAG5* amplification signature in all patients (HR, 1.96 [95% CI, 1.72-2.22]), as well as in the subclass of patients without *HER2* expression (HR, 2.17 [95% CI, 1.81-2.63]) and with *HER2* expression (HR, 1.52 [95% CI, 1.27-1.85]) (eFigure 3 in the Supplement).

Multivariable Cox regression models for 10-year BCSS confirmed that *SPAG5* transcript and SPAG5 protein expressions were associated with higher risk of death after controlling for other validated prognostic factors (METABRIC: HR, 1.96 [95% CI, 1.72-2.22]; adjusted *P* < .001; NUH-ESBC: HR, 1.68 [95% CI, 1.18-2.39]; adjusted *P* < .001; QBCFU: HR, 1.92 [95% CI, 1.11-3.35]; adjusted *P* = .02) (eTable 16 and eTable 17 in the Supplement).

After 2 weeks of preoperative endocrine therapy with aromatase inhibitors, mean (SD) *SPAG5* transcript expression was found to be significantly downregulated compared with pretreatment levels in 68 of 92 patients (74%) (0.23 [0.18] vs 0.34 [0.24]; *Z* = −5.24; *P* < .001). There was no statistically significant further reduction in the level of *SPAG5* trascript expression after 3 months compared with 2 weeks. In 73 of 92 patients with responding tumors (76%) in the NET cohort, a significant downregulation of mean (SD) *SPAG5* transcript expression in 68 of 73 patients (93%) occurred by 2 weeks compared with pretreatment levels (0.21 [0.16] vs 0.36 [0.24]; *P* < .001). However, in patients with nonresponding tumors, there was no significant change in *SPAG5* transcript levels by either 2 weeks or 3 months compared with pretreatment levels. By 3 months of treatment, median (IQR) *SPAG5* transcript was highly expressed in patients with nonresponding tumors compared with patients with responding tumors (0.36 [0.14-0.48] vs 0.18 [0.11-0.25]; Mann-Whitney *P* = .01) (eFigure 4 in the Supplement).

**Figure 2.**
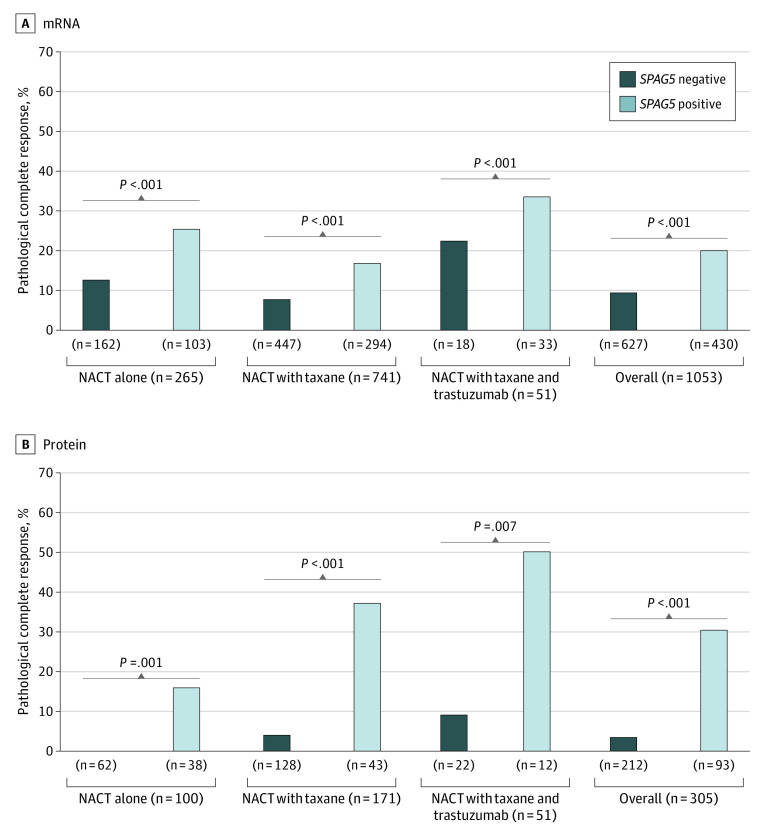
The Association of Pathological Complete Response With Sperm-Associated Antigen 5 (*SPAG5*) Expression After Receiving Anthracycline With or Without Taxane and Trastuzumab Neoadjuvant Chemotherapy (NACT)

After receiving NACT, patients with *SPAG5* transcript and SPAG5 protein expressions had higher pathological CR compared with patients without *SPAG5* transcript and SPAG5 protein expressions (MC-NACT cohort: 86 of 430 patients [20%] vs 58 of 627 patients [9%]; odds ratio [OR], 2.45 [95% CI, 1.71-3.51]; *P* < .001; NUH-LABC: 28 of 118 patients [24%] vs 9 of 221 patients [4%]; OR, 7.32 [95% CI, 3.33-16.22]; *P* < .001). Expression of *SPAG5* transcript or SPAG5 protein were associated with a higher pathological CR rates compared with no *SPAG5* transcript or SPAG5 protein expressions in patients who received either NACT alone (transcript: 26 of 103 patients [25%] vs 19 of 162 patients [12%]; *P* < .001; protein: 6 of 38 patients [16%] vs 0 of 62 patients [0%]; *P* < .001), or NACT with taxane (transcript: 50 of 294 patients [17%] vs 36 of 447 patients [8%]; *P* < .001; protein: 16 of 43 patients [37%] vs 5 of 128 patients [4%]; *P* < .001). In patients with *HER2* expression who received NACT with taxane and trastuzumab, patients with SPAG5 protein expression had statistically significantly higher pathological CR compared with patients without SPAG5 protein expression (6 of 12 patients [50%] vs 2 of 22 patients [9%]; *P* = .01) (Figure 2).

Notably, patients without *HER2* expression but with *SPAG5* transcript expression had 2-fold higher pathological CR after receiving NACT alone compared with those who received NACT with taxane (21 of 74 patients [28%] vs 38 of 245 patients [16%]; OR, 2.16 [95% CI, 1.17-3.98]; *P* = .01]. Strikingly, no patients without *HER2*, *SPAG5* transcript, or SPAG5 protein expressions who received NACT alone achieved pathological CR. However, patients with *HER2* and *SPAG5* transcript expressions who received NACT with taxane had similar pathological CR compared with patients who received trastuzumab in addition to NACT with taxane (12 of 48 patients [25%] vs 10 of 33 patients [30%]; *P* = .60) (Figure 2). Multivariable logistic regression models revealed that expressions of *SPAG5* in transcript (MC-NACT cohort: OR, 1.92 [95% CI, 1.01-3.64]; *P* = .05; MDACC cohort: OR, 0.03 [95% CI, 0-0.50]; *P* = .01) and SPAG5 protein (NUH-LABC cohort: OR, 23.03 [95% CI, 7.26-73.02]; *P* = 0.01) were independently associated with pathological CR (Table 1).

**Table 1.** Multivariable Logistic Regression Model Analysis for Pathological Complete Response After Neoadjuvant Chemotherapy Among Patients With Estrogen Receptor–Positive Breast Cancer

Variables	OR (95% CI)	*P* value
**Multivariable logistic regression models analysis for MC-NACT cohort **		
High *SPAG5* mRNA expression^a^	1.92 (1.01-3.64)	.047
*HER2* overexpression^a^	3.03 (1.34-6.88)	.008
Histological grade 3^b^	2.33 (1.25-4.34)	.008
PAM-50 molecular subclasses		
PAM50-LumA	1 [Reference]	.01
PAM50-LumB	0.24 (0.07-0.82)	
PAM50-HER2	0.97 (0.23-4.03)	
PAM50-Basal-like	0.72 (0.15-3.40)	
PAM50-Normal-like	0.32 (0.10-1.09)	
Received trastuzumab neoadjuvant chemotherapy^c^	0.66 (0.20-2.16)	.49
**Multivariable logistic regression models analysis for MDACC cohort**		
*SPAG5* transcript ^d^	0.03 (0.002-0.5)	.01
Ki67 expression^d^	1.33 (0.7-2.52)	.38
Histological grade 3^b^	2.7 (1.25-5.82)	.01
SPAG5*Ki67*grade	1.62 (1.06-2.46)	.03
Chemosensitivity prediction	4.96 (2.79-8.83)	<.001
Pathological CR prediction signature^a^	2.27 (1.2-4.27)	.01
LumB^e^	0.2 (0.07-0.58)	.003
**Multivariable logistic regression models analysis for NUH-LABC neoadjuvant cohort**		
High SPAG5 protein expression^a^	23.03 (7.26-73.02)	<.001
Histological grade^b^	2.37 (1.15-4.89)	.02
Patient age^f^	0.99 (0.96-1.01)	.26
*HER2* expression^d^	5.83 (1.97-17.22)	.001
Progesterone receptor^d^	0.23 (0.88-0.69)	.009
Received taxane neoadjuvant treatment^c^	1.52 (0.4-5.85)	.54

Abbreviations: CR, complete response; *HER2*, human epidermal growth factor receptor 2; MC-NACT, multicenter neoadjuvant anthracycline combination based chemotherapy; MDACC, MD Anderson Cancer Center taxane/anthracycline-based neo-adjuvant cohort; NUH-LABC, Nottingham University Hospital locally advanced breast cancer; OR, odds ratio; *SPAG5*, sperm-associated antigen 5.

^a^ Using low as the reference.

^b^ Using grade 1 or 2 as the reference.

^c^ Using not receiving the treatment as the reference.

^d^ Using no expression as the reference.

^e^ Using PAM50-LumA as the reference.

^f^ Continuous variable, OR is given per 1-year increase.

Of 57 patients with SPAG5 protein expression before NACT, 31 patients (54%) had been converted to no SPAG5 protein expression in their residual post-NACT surgical specimens after receiving NACT. Among 185 patients without SPAG5 protein expression before NACT, 13 patients (7%) had SPAG5 protein expression in the residual tissue (McNemar *P* = .01).

**Figure 3.**
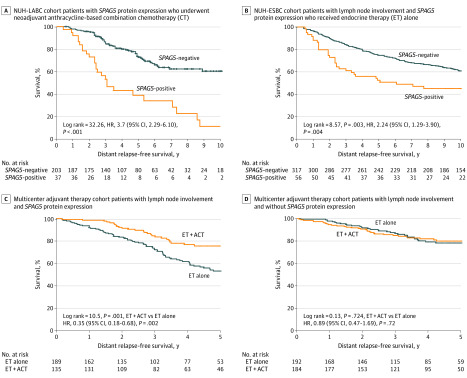
Distant Relapse–Free Survival Associated With Sperm-Associated Antigen 5 (*SPAG5*) Expression in Patients with Estrogen Receptor–Positive Breast Cancer ACT indicates anthracycline combination chemotherapy; ESBC, early stage breast cancer; HR, hazard ratio; and NUH, Nottingham University Hospital locally advanced breast cancer.

In patients without *HER2* overexpression who received NACT followed by 5-year adjuvant endocrine therapy, we observed a similar 5-year DRFS among patients with and without *SPAG5* transcript expression (MDACC: HR, 1.18 [95% CI, 0.74-1.88]; *P* = .50) and among patients with and without SPAG5 protein expression (NUH-LABC: HR, 0.83 [95% CI, 0.48-1.42]; *P* = .49) (eFigure 5 in the Supplement). However, in patients with residual disease after NACT, SPAG5 protein expression was associated with shorter 5-year DRFS compared with no SPAG5 protein expression (NUH-LABC: HR, 3.73 [95% CI, 2.29-9 6.10]; *P* < .001) (Figure 3A).

Among patients without lymph node involvement, SPAG5 protein expression, compared with no SPAG5 protein expression, was associated with shorter DRFS in patients who did not receive systemic adjuvant therapy (HR, 1.82 [95% CI, 1.27-1.59]; *P* = .001) or received adjuvant endocrine therapy alone (HR, 2.52 [95% CI, 1.53-4.16]; *P* < .001) (eFigure 5 in the Supplement). Among patients who received adjuvant endocrine therapy and chemotherapy, there was no significant difference in DRFS between patients with or without SPAG5 protein expression (HR, 0.33 [95% CI, 0.04-2.67]; *P* = .30). Among patients without lymph node involvement, those with *SPAG5* transcript expression who received adjuvant endocrine therapy with chemotherapy had longer 5 year DRFS (45 of 51 patients [89%]) compared with those who received adjuvant endocrine therapy alone (97 of 145 patients [67%]) or did not receive adjuvant therapy (125 of 205 patients [61%]). There were no significant differences in DRFS among patients without *SPAG5* transcript expression (eFigure 6 in the Supplement).

In patients with lymph node involvement, SPAG5 protein expression was associated with shorter DRFS in those who received adjuvant endocrine therapy alone compared with those without SPAG5 protein expression (NUH-ESBC: HR, 2.24 [95% CI, 1.29-3.90]; *P* = .004) (Figure 3B). Among patients with lymph node involvement who received adjuvant endocrine therapy with chemotherapy, there was no significant difference in DRFS between patients with or without SPAG5 protein expression (NUH-ESBC: HR, 1.00 [95% CI, 0.61-1.64]; *P* > .99) (eFigure 6 in the Supplement). Patients with *SPAG5* transcript expression and lymph node involvement who received adjuvant endocrine therapy with chemotherapy had longer 5-year DRFS (144 patients [76%]) compared with those who received adjuvant endocrine therapy alone (104 patients [54%]), whereas there was no significant difference among patients without *SPAG5* transcript expression and with lymph node involvement (Figure 3C and D).

A multivariate Cox regression model for 5-year DRFS confirmed that *SPAG5* transcript (HR. 2.59 [95% CI, 1.69-3.97]; *P* < .001) or SPAG5 protein (HR, 1.68 [95% CI, 1.18-2.93]; *P* = .004) were associated with poor prognosis after controlling for adjuvant endocrine therapy and other validated prognostic factors. The interaction-term of *SPAG5* transcript expression with chemotherapy was statistically significant (HR, 0.33 [95% CI, 0.17-0.67]; *P* = .002) (Table 2).

**Table 2.** Multivariable Cox Regression Models Analysis of 5-Year Distant Relapse Free Survival Among Patients With Estrogen Receptor–Positive Breast Cancer

Variables	HR (95% CI)	*P* value
**MC-AT cohort **		
Overall model for *SPAG5* transcript expression		
*SPAG5* mRNA^a^	2.59 (1.69-3.97)	<.001
*MKI67* mRNA^a^	0.99 (0.49-1.71)	.77
Lymph node status^b^	1.81 (1.01-1.29)	.001
Tumor size, cm		
≤2	1 [Reference]	.03
>2	1.46 (1.04-2.05)	
Progesterone receptor^c^	0.63 (0.43-0.91)	.01
Histologic grade		
Low	1 [Reference]	.005
Intermediate	2.59 (1.34-5.01)	
High	3.18 (1.59-6.36)	
Size	1.01 (1.007-1.015)	<.001
Endocrine and chemotherapies		
No systemic therapy	1 [Reference]	.004
Endocrine therapy alone	0.57 (0.27-1.18)	
Endocrine therapy with chemotherapy	0.29 (0.13-0.66)	
Anthracycline chemotherapy*SPAG5	0.33 (0.17-0.67)	.002
*SPAG5* vs PAM-50 molecular classes		
PAM-50 molecular subclasses		
PAM50-LumA	1 [Reference]	.09
PAM50-LumB	1.58 (0.91-2.72)	
PAM50-*HER2*	1.41 (0.85-2.34)	
PAM50-basal-like	0.86 (0.62-1.18)	
PAM50-normal-like	1.18 (0.85-1.64)	
*SPAG5* mRNA overexpression^c^	1.85 (1.26-2.70)	.002
**NUH-ESBC cohort **		
Overall model for SPAG5 protein expression^c^		
SPAG5 protein expression	1.68 (1.18-2.93)	.004
Ki67 protein expression	0.71 (0.47-1.06)	.09
TOP2A protein expression	0.91 (0.65-1.27)	.57
Tumor size^d^	1.59 (1.23-2.07)	<.001
Lymph node status		
Negative	1 [Reference]	<.001
Positive	1.72 (1.44-2.05)	
Histological grade		
Low or intermediate	1 [Reference]	<.001
High	2.63 (2.10-3.30)	
*HER2* overexpression^c^	1.68 (1.22-2.32)	.004
Anthracycline chemotherapy^e^	0.68 (0.48-0.94)	.02
Endocrine therapy^e^	0.76 (0.56-1.03)	.08

Abbreviations: *HER2*, human epidermal growth factor receptor 2; HR, hazard ratio; MC-AT, multicenter adjuvant therapy; *SPAG5*, sperm-associated antigen 5.

^a^ Using low as the reference.

^b^ Using no involvement as the reference.

^c^ Using no expression as the reference.

^d^ Continuous variable, HR is given per 1-cm increase.

^e^ Using not receiving the treatment as the reference.

## Discussion

SPAG5 is a microtubule-associated protein required for mitotic spindle formation and chromosome segregation, and its depletion causes multipolar spindle formation, aneuploidy, and cell death.^17^ In this cohort study, we have validated *SPAG5* as a biomarker associated with endocrine therapy and chemotherapy in estrogen receptor–positive breast cancers, in agreement with previous studies in breast,^8,18^ lung,^19^ and cervical cancers.^17^ Our data also suggested that *SPAG5* could be an important genetic driver in estrogen receptor–positive breast cancer,^8,9^ as *SPAG5* dysregulation could contribute to high chromosomal instability and aneuploidy, which are hallmarks of malignant cells and confer susceptibility to chemotherapy. Unlike most currently used clinicopathological and multigene tests, *SPAG5* has also potential as a predictive and monitoring tool for endocrine therapy and chemotherapy response. Moreover, *SPAG5* downregulation after neoadjuvant endocrine therapy and NACT is associated with the clinical outcome of the adjuvant endocrine therapy. *SPAG5* expression could be associated with differential sensitivity to anthracycline and taxane. These findings are in agreement with previous studies that suggested anthracycline works best in tumors with higher proliferation and chromosomal instability,^20,21^ whereas endocrine therapy and taxane work best in chromosomally stable low proliferative breast cancer.^15,22^

Studies from 2013^23^ and 2008^24^ have shown that combining chemotherapy, and PI3K or mTOR inhibitors with endocrine therapy restores endocrine therapy responsiveness. In this study and our previous work,^8^ *SPAG5* transcript and SPAG5 protein expressions were associated with factors that have been reported to be associated with endocrine therapy resistance and could be a target for novel therapeutic strategies in endocrine therapy resistance (eg, *FOXM1* [OMIM 602341], *mTOR* [OMIM 601231], and *ESPL1* [OMIM 604143] and their corresponding proteins).^25^ *SPAG5* downregulation has been reported to alter mTOR activity and eventually influence the apoptosis.^17^ Experiments in cervical cancer have demonstrated that *SPAG5* exerts a vital moderating effect on taxol treatment by switching apoptosis off and on via mTOR.^17^ Moreover, downregulation of *SPAG5* has been demonstrated after treatment with endocrine therapy,^15^ PI3K inhibitor, mTOR inhibitors, and trastuzumab when combined with taxol.^23,24^ Therefore, *SPAG5* may not only monitor the response to endocrine therapy, but also could be used to select patients who would benefit from additional therapeutic drugs.

Examination of most breast cancer prognostic tests or assays shows that the proliferation cassette is common to all.^26^ However, most of these tests do not predict the therapeutic benefit from chemotherapy or endocrine therapy_._ For instance, although the Oncotype DX test (Oncotype IQ) is informative, the decision regarding systemic therapy remains challenging for clinicians for more than 50% of patients with estrogen receptor–positive early breast cancer.^26,27^ Moreover, the routine use of Ki67 in clinical practice has been limited by the lack of its standardization assessment^28^ and by its lack of success in treatment decision-making in several clinical trials.^29,30^

### Limitations

This study has some limitations. The main limitation of our study is that it was a retrospective observational study. Although it accumulated data from a large number of unselected patients, the patients were neither standardized nor uniformly treated. Therefore, validation of our results in a prospective clinical trial is recommended. Moreover, there is no direct comparison of *SPAG5* with Oncotype DX.

## Conclusions

This cohort study found that *SPAG5* transcript and SPAG5 protein expressions were associated with therapeutic response. This gene and its associated protein could potentially be used to match and monitor effective drugs with individual patients.

## Notes

Supplement. **eAppendix.** Supplementary Methods **eTable 1.** Table of Antibodies and Optimisation Conditions Used to Immunohistochemically Profile the Nottingham University Hospitals–Based Cohorts **eTable 2.** Clinicopathological Characteristics of Molecular Taxonomy of Breast Cancer International Consortium Cohort **eTable 3.** Clinicopathological Characteristics of The Cancer Genome Atlas-Breast Cancer Project Cohort **eTable 4.** Clinicopathological Characteristics of the Swegene Cohort **eTable 5.** Characteristics of Patients in the Nottingham University Hospital Early Stage Breast Cancer Cohort **eTable 6.** Characteristics of Patients in the Neoadjuvant Endocrine Therapy Cohort **eTable 7.** Gene Expression Platforms of Multicenter Neoadjuvant Anthracycline-Based Combination Chemotherapy Cohort **eTable 8.** Characteristics of Patients in the Multicenter Neoadjuvant Anthracycline-Based Combination Chemotherapy Cohort **eTable 9.** Characteristics of Patients in the Nottingham University Hospital Locally Advanced Breast Cancer Cohort **eTable 10.** Clinicopathological Characteristics in the MD Anderson Cancer Center Cohort **eTable 11.** Characteristics of Patients in the Multicenter Adjuvant Therapy Cohort **eTable 12.** Gene Expression Platforms of Multicenter Adjuvant Therapy Cohort **eTable 13.** Association of *SPAG5* mRNA Expression and Clinicopathologic Variables in the Molecular Taxonomy of Breast Cancer International Consortium Cohort **eTable 14.** Association of *SPAG5* mRNA Expression and Clinicopathologic Variables in the Swegene Cohort **eTable 15.** Clinicopathological Association of SPAG5 Protein Expression in the Nottingham Historical Early Stage Breast Cancer Cohort **eTable 16.** Multivariable Cox Regression Models Analysis for 5-Year Overall Survival in the Nottingham University Hospital Early Stage Breast Cancer Cohort **eTable 17.** Multivariable Cox Regression Models Analysis for 5-Year Overall Survival in Queensland Breast Cancer Follow-Up Cohort **eFigure 1.** Clinical Outcome of *SPAG5* Copy Number Variants and Transcript Expression and SPAG5 Protein Expression in the Estrogen Receptor–Positive Breast Cancer **eFigure 2.** Clinical Outcome of *SPAG5* Transcript and SPAG5 Protein Expression in the Molecular Taxonomy of Breast Cancer International Consortium and Nottingham University Hospital Early Stage Breast Cancer Cohorts **eFigure 3.** Clinical Outcome of *SPAG5* Amplification Transcriptomic Signature **eFigure 4.** *SPAG5* Transcript Expression and Clinical Response to Neoadjuvant Endocrine Therapy **eFigure 5.** *SPAG5* Transcript Expression and Clinical Response to Neoadjuvant Endocrine Therapy **eFigure 6.** Kaplan-Meier Curves Showing the Outcomes of the Received Adjuvant Systemic Therapy on Distant Relapse Free Survival in Patients With Low and High *SPAG5* Transcript, Without Lymph Node Involvement and High or Low *SPAG5* Transcript in the Multicenter Adjuvant Therapy Cohort
