# Supplementary material for: Association of Sperm-Associated Antigen 5 and Treatment Response in Patients With Estrogen Receptor–Positive Breast Cancer
Source: JAMA Netw Open. 2020 Jul 7;3(7):e209486. doi: 10.1001/jamanetworkopen.2020.9486 (PMC7341179; doi:10.1001/jamanetworkopen.2020.9486)
Supplement: Supplement. — eAppendix. Supplementary Methods eTable 1. Table of Antibodies and Optimisation Conditions Used to Immunohistochemically Profile the Nottingham University Hospitals–Based Cohorts eTable 2. Clinicopathological Characteristics of Molecular Taxonomy of Breast Cancer International Consortium Cohort eTable 3. Clinicopathological Characteristics of The Cancer Genome Atlas-Breast Cancer Project Cohort eTable 4. Clinicopathological Characteristics of the Swegene Cohort eTable 5. Characteristics of Patients in the Nottingham University Hospital Early Stage Breast Cancer Cohort eTable 6. Characteristics of Patients in the Neoadjuvant Endocrine Therapy Cohort eTable 7. Gene Expression Platforms of Multicenter Neoadjuvant Anthracycline-Based Combination Chemotherapy Cohort eTable 8. Characteristics of Patients in the Multicenter Neoadjuvant Anthracycline-Based Combination Chemotherapy Cohort eTable 9. Characteristics of Patients in the Nottingham University Hospital Locally Advanced Breast Cancer Cohort eTable 10. Clinicopathological Characteristics in the MD Anderson Cancer Center Cohort eTable 11. Characteristics of Patients in the Multicenter Adjuvant Therapy Cohort eTable 12. Gene Expression Platforms of Multicenter Adjuvant Therapy Cohort eTable 13. Association of SPAG5 mRNA Expression and Clinicopathologic Variables in the Molecular Taxonomy of Breast Cancer International Consortium Cohort eTable 14. Association of SPAG5 mRNA Expression and Clinicopathologic Variables in the Swegene Cohort eTable 15. Clinicopathological Association of SPAG5 Protein Expression in the Nottingham Historical Early Stage Breast Cancer Cohort eTable 16. Multivariable Cox Regression Models Analysis for 5-Year Overall Survival in the Nottingham University Hospital Early Stage Breast Cancer Cohort eTable 17. Multivariable Cox Regression Models Analysis for 5-Year Overall Survival in Queensland Breast Cancer Follow-Up Cohort eFigure 1. Clinical Outcome of SPAG5 Copy Number Variants and Transcript E [file jamanetwopen-3-e209486-s001.pdf]

Supplementary Online Content

Abdel-Fatah TMA, Ball GR, Thangavelu PU, et al. Association of sperm-associated antigen 5 and treatment response in patients with estrogen receptor–positive breast cancer. *JAMA Netw Open*. 2020;3(7):e209468. doi:10.1001/jamanetworkopen.2020.9486

eAppendix. Supplementary Methods

eTable 1. Table of Antibodies and Optimisation Conditions Used to Immunohistochemically Profile the Nottingham University Hospitals–Based Cohorts

eTable 2. Clinicopathological Characteristics of Molecular Taxonomy of Breast Cancer International Consortium Cohort

eTable 3. Clinicopathological Characteristics of The Cancer Genome Atlas-Breast Cancer Project Cohort

eTable 4. Clinicopathological Characteristics of the Swegene Cohort

eTable 5. Characteristics of Patients in the Nottingham University Hospital Early Stage Breast Cancer Cohort

eTable 6. Characteristics of Patients in the Neoadjuvant Endocrine Therapy Cohort

eTable 7. Gene Expression Platforms of Multicenter Neoadjuvant Anthracycline-Based Combination Chemotherapy Cohort

eTable 8. Characteristics of Patients in the Multicenter Neoadjuvant Anthracycline-Based Combination Chemotherapy Cohort

eTable 9. Characteristics of Patients in the Nottingham University Hospital Locally Advanced Breast Cancer Cohort

eTable 10. Clinicopathological Characteristics in the MD Anderson Cancer Center Cohort

eTable 11. Characteristics of Patients in the Multicenter Adjuvant Therapy Cohort

eTable 12. Gene Expression Platforms of Multicenter Adjuvant Therapy Cohort

eTable 13. Association of *SPAG5* mRNA Expression and Clinicopathologic Variables in the Molecular Taxonomy of Breast Cancer International Consortium Cohort

eTable 14. Association of *SPAG5* mRNA Expression and Clinicopathologic Variables in the Swegene Cohort

eTable 15. Clinicopathological Association of SPAG5 Protein Expression in the Nottingham Historical Early Stage Breast Cancer Cohort

© 2020 Abdel-Fatah TMA et al. *JAMA Network Open.*

eTable 16. Multivariable Cox Regression Models Analysis for 5-Year Overall Survival in the Nottingham University Hospital Early Stage Breast Cancer Cohort

eTable 17. Multivariable Cox Regression Models Analysis for 5-Year Overall Survival in Queensland Breast Cancer Follow-Up Cohort

eFigure 1. Clinical Outcome of *SPAG5* Copy Number Variants and Transcript Expression and SPAG5 Protein Expression in the Estrogen Receptor–Positive Breast Cancer

eFigure 2. Clinical Outcome of *SPAG5* Transcript and SPAG5 Protein Expression in the Molecular Taxonomy of Breast Cancer International Consortium and Nottingham University Hospital Early Stage Breast Cancer Cohorts

eFigure 3. Clinical Outcome of *SPAG5* Amplification Transcriptomic Signature

eFigure 4. *SPAG5* Transcript Expression and Clinical Response to Neoadjuvant Endocrine Therapy

eFigure 5. *SPAG5* Transcript Expression and Clinical Response to Neoadjuvant Endocrine Therapy

eFigure 6. Kaplan-Meier Curves Showing the Outcomes of the Received Adjuvant Systemic Therapy on Distant Relapse Free Survival in Patients With Low and High *SPAG5* Transcript, Without Lymph Node Involvement and High or Low *SPAG5* Transcript in the Multicenter Adjuvant Therapy Cohort

This supplementary material has been provided by the authors to give readers additional information about their work.

© 2020 Abdel-Fatah TMA et al. *JAMA Network Open.*

eAppendix. Supplementary Methods

Nottingham University Hospital early stage breast cancer cohort (n=2500)

This cohort of BC include 2500 patients (age>71 years) who were diagnosed and treated uniformly between 1986 and 2006 at the Nottingham City Hospital (NCH), Nottingham, UK. Patients within the good prognosis group (Nottingham Prognostic Index (NPI) <3.4) did not receive systemic adjuvant therapy. ER-positive patients with moderate or poor NPI were offered tamoxifen ET therapy for 5-years. Pre-menopausal patients within the moderate and poor prognosis groups that were treated before 2000 were candidates for CMF CT (cyclophosphamide 750 mg m^{-2}, methotrexate 50 mg m^{-2} and 5-fluorouracil 1 g m^{-2}, on day 1 of a 21-day cycle.) whereas those treated after 2000 were received anthracycline based combination (AC) CT. None of the human epidermal growth factor receptor-2 (HER2) overexpression patients received trastuzumab. Clinical data were maintained on a prospective basis.

University of Queensland, Australia cohort

The IHC of SPAG5 protein has been externally validated at University of Queensland centre using Queensland breast cancer follow-up (QFU) cohort, which comprises TMAs of 298 ER+ invasive breast carcinomas (sampled in duplicate at a minimum) with associated clinical data, including OS up to 35 years post-diagnosis (median follow-up 14.1 years, range 0.03 -41.75 years). The samples were collected from the Royal Brisbane and Women's Hospital (RBWH) between 1987 and 1994. Pathological and clinical data for these patients were obtained from a combination of clinical diagnostic pathology reports (Pathology Queensland), the Queensland Cancer Registry and internal histopathology review (SRL). The use of samples and clinical data for this study were approved by human research ethics committees of the University of Queensland and RBWH.

1. Field, S., et al., Novel highly specific anti-periostin antibodies uncover the functional importance of the fascilin 1-1 domain and highlight preferential expression of periostin in aggressive breast cancer. Int J Cancer, 2016. 138(8): p. 1959-70. 2.
2. Junankar, S., et al., ID4 controls mammary stem cells and marks breast cancers with a stem cell-like phenotype. Nat Commun, 2015. 6: p. 6548. 3.
3. Al-Ejeh, F., et al., Meta-analysis of the global gene expression profile of triple-negative breast cancer identifies genes for the prognostication and treatment of aggressive breast cancer. Oncogenesis, 2014. 3: p. e124.

© 2020 Abdel-Fatah TMA et al. *JAMA Network Open.*

eTable 1. Table of Antibodies and Optimisation Conditions Used to Immunohistochemically Profile the Nottingham University Hospitals–Based Cohorts

Antigen	Antibody	Clone	Source	Antigen Retrieval	Dilution / Incubation Time	Distribution	Scoring system	Cut-offs
p53	Mouse MAb anti p53	DO7	Novocastra	Citrate pH6	1: 50 60 min	Nuclear	% of positive cells	≤20% (negative) >20% (High)
Bcl2	Mouse MAb anti-Bcl2	124	Dako-Cytomation	Citrate pH6	1:100 60 min	Cytoplasm	% of positive cells	>10% (positive
BRCA 1	BRCA1	MS110	Calbiochem	Citrate pH6	1:100 60 min	Nuclear	% of positive cells	<25% (negative)
ATM	Rabbit MAb anti-ATM	Y170	Abcam	Citrate pH6	1:100 18 hours	Nuclear	% of positive cells	<25% (negative)
p27	anti-p27	SX53G8	Dako-Cytomation	Citrate pH6	1:50 60 min	Nuclear	% of positive cells	≥10% (positive)
Vimentin	Mouse MAb anti-vimentin	Vim 3B4	Dako-Cytomation	Citrate pH6	1:250 60 min	Cytoplasm	% of positive cells	≥10% (positive)
Bax	Rabbit anti-Bax	Polyclonal	Abcam	Citrate pH6	1:1000 60 min	Cytoplasm	% positive cells	≥10% (positive)
ER	Mouse MAb anti-ER-α	SP1	Dako-Cytomation	Citrate pH6	1:150 30 min	Nuclear	Allred score	≥3 (positive)
ER	Mouse MAb anti-ER-α	EP1	Dako-Cytomation	Citrate pH6	1:80 30 min	Nuclear	% positive cells	≥1% positive
PR	Mouse MAb anti-PR	PgR636	Dako-Cytomation	Citrate pH6	1:125 30 min	Nuclear	% positive cells	≥1% positive
EGFR	Mouse MAb anti-EGFR	31G7	Zymed Laboratories	Proteinase K, 370C for 8 min	1:30 60 min	Membrane	0-3 as HER2	0 or +1 (negative) +2 or +3 (positive)
CK14	Mouse MAb anti-Ck14	LL002	Novocastra	Citrate pH6	1:40 60 min	Cytoplasm	% of positive cells	≥10% (positive)
Ck5/6	Mouse MAb anti-Ck5/6	D5/161 B4	Dako-Cytomation	EDTA pH8	1:100 60 min	Cytoplasm	% of positive cells	≥10% (positive)

© 2020 Abdel-Fatah TMA et al. *JAMA Network Open.*

HER2	Rabbit antihuman c-erbB2	polyclonal	Dako-Cytomation	None	1:400 60 min	Membrane	See text	See text
Ki67	Mouse MAb anti-Ki-67	MIB1	Dako-Cytomation	Citrate pH6	1:300 60 min	Nuclear	% of positive cells	0-30% (low) >30% (high)
TOP2A	Mouse MAb TOP2A	KiS1	Dako-Cytomation	Citrate pH6	1:100 60 min	Nuclear	% of positive cells	>25% (positive)
p21	Mouse MAb anti-p21	SW118	Dako-Cytomation	Citrate pH6	1:50 60 min	Nuclear	% of positive cells	≥10% (positive)
MDM2	Mouse MAb anti-MDM2	1B10	Novocastra	Citrate pH6	1:200 60 min	Nuclear	% of positive cells	≥10% (High)
MDM4	Affinity purified rabbit anti-HdmX/MDM4	IHC-00108	Bethyl Labs	Citrate pH6	1:100 60 min	Nuclear	% of positive cells	0-20% (Low) >20% (High)
HER3	Mouse MAb anti-HER3	RTJ1	Novocastra	Citrate pH6	1:20 60 min	Cytoplasm and Membrane	H score	H score <150
HER4	Rabbit antihuman c-erbB4	polyclonal	Neo Marker	None	6:4 60 min	Cytoplasm	H score	H score <100
P-cadherin	Mouse MAb anti-P-cadherin	Clone 56	BD Bioscience	None	1:200 60 min	cytoplasm	% of positive cells	>5% positive
E-cadherin	Mouse MAb anti-E-cadherin	HERCD-1	Zymed Laboratories	Citrate pH6	1:100 60 min	Membrane	H score	H score ≤100
SPAG5	Rabbit anti-SPAG5	polyclonal	Sigma-Aldrich	Citrate pH6	1:50 60 min	Cytoplasmic	% of positive cells	>10% (positive)

All sections were pre-treated with microwave antigen retrieval using 0·1% citrate buffer (pH 6) except for HER2 (no pre-treatment) and EGFR (pre-treated with protease for 10 minutes). MAb: Monoclonal antibody; MDM2: murine double minute 2; MDM4: murine double minute 4; ATM: ataxia telangiectasia mutated; BRCA1: BC 1, ER: oestrogen receptor; PR: progesterone receptor; CK: cytokeratin; EGFR: epidermal growth factor; TOP2A: Topoisomerase II alpha; HAGE: helicase Antigen, HER2 (ERBB2): v-erb-b2 erythroblastic leukemia viral oncogene homolog 2, neuro/glioblastoma derived oncogene homolog (avian), HER3 (ERBB3): v-erb-b2 erythroblastic leukemia viral oncogene homolog 3 (avian), HER4 (ERBB4): human

© 2020 Abdel-Fatah TMA et al. *JAMA Network Open.*

epidermal receptor 4 , Bcl2: B-cell CLL/lymphoma 2, Bax: BCL2-associated X protein , SPAG5: sperm associated antigen 5, KIF2C: kinesin family member 2C.

© 2020 Abdel-Fatah TMA et al. *JAMA Network Open.*

eTable 2. Clinicopathological Characteristics of Molecular Taxonomy of Breast Cancer International Consortium Cohort

Features		Number	Percentage
SPAG5	Overexpression (+)	658	43.9
	Low	840	56.1
HER2 (+) Overexpression/amplification	No	1383	92.3
	Yes	115	7.7
PR status	Negative	515	34.4
	Positive	983	65.6
Histological Grade	Low (G1)	158	11.0
	Intermediate (G2)	708	49.3
	High (G3)	568	37.9
	Unknown	64	4.3
Molecular PAM-50 intrinsic subtype	Basal-like	56	3.7
	Her2-enriched	111	7.4
	Luminal A	694	46.3
	Luminal B	471	31.4
	Normal	160	10.7
	Unknown	6	0.4
Tumor size	≤1 cm	67	4.5
	1-2cm	418	27.9
	2-5cm	939	62.7
	>5cm	61	4.1
	Unknown	13	0.9
Lymph node Metastases	Negative	816	54.5
	Positive	682	45.5
Adjuvant systemic therapy regimen	No systemic therapy	387	25.9
	Endocrine therapy alone	968	64.6
	Endocrine therapy + chemotherapy	143	9.5
Death	No	1159	77.4
	Yes	339	22.6

© 2020 Abdel-Fatah TMA et al. *JAMA Network Open.*

eTable 3. Clinicopathological Characteristics of The Cancer Genome Atlas-Breast Cancer Project Cohort

Features		Number	Percentage
SPAG5	Overexpression (+)	162	42.5
	Low	223	57.5
HER2 (+) Overexpression/amplification	No	279	73.2
	Yes	66	17.4
	unknown	36	9.4
PR status	Negative	61	16.0
	Positive	318	83.5
	unknown	2	0.5
Lymph node Metastases	Negative	183	48.0
	Positive	193	50.7
	unknown	5	1.3
Adjuvant systemic therapy regimen	No systemic therapy	221	58.0
	Endocrine therapy alone	53	13.9
	Endocrine therapy + chemotherapy	100	26.2
	unknown	7	1.8
Trastuzumab	No	365	95.8
	Yes	16	4.2
Death	No	1159	93.7
	Yes	24	6.3

© 2020 Abdel-Fatah TMA et al. *JAMA Network Open.*

eTable 4. Clinicopathological Characteristics of the Swegene Cohort

Features		Number	Percentage
SPAG5	Overexpression (+)	89	39.2
	Low	138	60.8
PR status	Negative	49	21.6
	Positive	174	76.6
	Unknown	4	1.8
Histological Grade	Low (G1)	22	9.7
	Intermediate (G2)	77	33.9
	High (G3)	40	17.6
	Unknown	88	38.8
Molecular PAM-50 intrinsic subtype	Basal-like	5	2.2
	Her2-enriched	10	4.4
	Luminal A	89	39.2
	Luminal B	64	28.2
	Normal	24	10.6
	Unknown	35	15.4
Death	No	128	56.4
	Yes	339	41.4
	unknown	5	2.2

© 2020 Abdel-Fatah TMA et al. *JAMA Network Open.*

eTable 5. Characteristics of Patients in the Nottingham University Hospital Early Stage Breast Cancer Cohort

Features		Number	Percentage
SPAG5	Overexpression (+)	1956	78.2
	Low	321	12.9
	Unknown	223	8.9
HER2 (+) Overexpression/amplification	NO	2105	84.2
	Yes	168	6.7
	Unknown	227	9.1
Lymph node Metastases	Negative	1590	63.6
	Positive	893	35.7
	unknown	17	0.7
PR status	Negative	810	32.4
	Positive	1423	56.9
	Unknown	267	10.7
Histological Grade	Low (G1)	550	22.0
	Intermediate (G2)	1118	44.7
	High (G3)	813	32.5
	Unknown	19	0.8
Tumor size (cm)	<=2	1450	58
	>2	935	37.4
	Unknown	115	4.6
Adjuvant therapy			
Adjuvant systemic therapy regimen	No systemic therapy	1175	47.0
	Endocrine therapy alone	1050	42.0
	Endocrine therapy + chemotherapy	275	11.0
Adjuvant Endocrine therapy (n=1050)	Tamoxifen	1030	98.0
	Aromatase Inhibitors	20	2.0
Chemotherapy (n=275)	Anthracycline based only	115	21.7
	Anthracycline based+ Taxane	348	65.8
	CMF	66	12.5
Trastuzumab	Yes	0	0
	No	2500	100
Distant relapse	No	1860	74.4
	Yes	640	25.6

© 2020 Abdel-Fatah TMA et al. *JAMA Network Open.*

Neo-adjuvant endocrine therapy cohort:

total of 101 patients from a consecutive series of postmenopausal women who presented to Western General Hospital in Edinburgh with large primary histologically confirmed oestrogen receptor (ER) –rich (Allred scores 6 to 8) invasive breast cancer and who were recruited between 2003 and 2011fulfilled the requirements to be included in this study. All patients gave informed consent and the study was approved by the local regional ethics committee (2001/8/80and 2001/8/81). Patients were treated with letrozole (Femara, 2.5 mg; Novartis Pharma AG, Basel, Switzerland) daily for at least 3 months.

eTable 6. Characteristics of Patients in the Neo-Adjuvant Endocrine Therapy Cohort

Features		Number	Percentage
ER (IHC Allred score)	6	2	2.0
	7	15	14.9
	8	72	71.3
	unknown	12	11.9
ER (H-score)	mean	223.4	
	range	112-292	
Histological Grade	Low (G1)	11	11.9
	Intermediate (G2)	62	61.4
	High (G3)	16	15.8
	Unknown	12	11.9
Molecular intrinsic subtype	Basal-like	0	0
	Her2-enriched	0	0
	Luminal A	71	70.3
	Luminal B	13	12.9
	Normal	0	0
	Unknown	17	16.8
Lymph node metastases	NO	65	64.4
	Yes	24	23.7
	unknown	12	11.9
T Stage	T1	6	5.9
	T2	56	55.5
	T3	9	8.9
	T4	18	17.8
	Unknown	12	11.9
Distant metastases	no	81	80.2
	yes	5	5.0
	unknown	15	14.8

© 2020 Abdel-Fatah TMA et al. *JAMA Network Open.*

eTable 7. Gene Expression Platforms of Multicenter Neoadjuvant Anthracycline-Based Combination Chemotherapy Cohort

Reference	Access Number	Data	Chip	Platform	Number of patients	percentage
1	GSE20194	Affymetrix	HG-U133A	GPL96	151	14.1
2	GSE20271	Affymetrix	HG-U133A	GPL96	98	9.1
3	GSE25066	Affymetrix	HG-U133A	GPL96	299	27.9
4	GSE37946	Affymetrix	HG-U133A	GPL96	18	1.7
5	GSE42822	Affymetrix	HG-U133A	GPL96	38	3.5
6	GSE32646	Affymetrix	HG-U133_Plus_2	GPL570	71	6.6
7	GSE50948	Affymetrix	HG-U133_Plus_2	GPL570	52	4.8
8	GSE41998	Affymetrix	HG-U133A_2	GPL571	108	10.1
9	GSE6861	Affymetrix	HG-U133_X3P	GPL1352	37	3.4
10	GSE34138	ILLUMINA	HumanWG-6 v3.0	GPL6884	120	11.2
11	GSE22226	Agilent	Agilent-014850	GPL1708	81	7.5

1. Popovici V, Chen W, Gallas BG, et al. Effect of training-sample size and classification difficulty on the accuracy of genomic predictors. Breast Cancer Res; 2010; 12(1):R5
2. Tabchy A, Valero V, Vidaurre T, et al. Evaluation of a 30-gene paclitaxel, fluorouracil, doxorubicin, and cyclophosphamide chemotherapy response predictor in a multicenter randomized trial in breast cancer. Clin Cancer Res. 2010; 16(21):5351-61
3. Hatzis C, Pusztai L, Valero V, et al. A genomic predictor of response and survival following taxane-anthracycline chemotherapy for invasive breast cancer. JAMA. 2011; 305(18):1873-81
4. Liu JC, Voisin V, Bader GD, et al. Seventeen-gene signature from enriched Her2/Neu mammary tumor-initiating cells predicts clinical outcome for human HER2+:ERα- breast cancer. Proc Natl Acad Sci U S A. 2012; 109(15):5832-7
5. Shen K, Qi Y, Song N, et al. Cell line derived multi-gene predictor of pathologic response to neoadjuvant chemotherapy in breast cancer: a validation study on US Oncology 02-103 clinical trial. BMC Med Genomics. 2012;5:51
6. Miyake T, Nakayama T, Naoi Y, et al. GSTP1 expression predicts poor pathological complete response to neoadjuvant chemotherapy in ER-negative breast cancer. Cancer Sci. 2012; 103(5):913-20.
7. Prat A, Bianchini G, Thomas M, et al. Research-based PAM50 subtype predictor identifies higher responses and improved survival outcomes in HER2-positive breast cancer in the NOAH study. Clin Cancer Res. 2014; 20(2):511-21

© 2020 Abdel-Fatah TMA et al. *JAMA Network Open.*

8. Horak CE, Pusztai L, Xing G, et al. Biomarker analysis of neoadjuvant doxorubicin/cyclophosphamide followed by ixabepilone or Paclitaxel in early-stage breast cancer. Clin Cancer Res. 2013; 19(6):1587-95
9. Bonnefoi H, Potti A, Delorenzi M, et al. Validation of gene signatures that predict the response of breast cancer to neoadjuvant chemotherapy: a substudy of the EORTC 10994/BIG 00-01 clinical trial. Lancet Oncol. 2007; 8(12):1071-1078.
10. de Ronde JJ, Lips EH, Mulder L, et al. SERPINA6, BEX1, AGTR1, SLC26A3, and LAPTM4B are markers of resistance to neoadjuvant chemotherapy in HER2-negative breast cancer. Breast Cancer Res Treat. 2013; 137(1):213-23.
11. Esserman LJ, Berry DA, Cheang MC, et al. Chemotherapy response and recurrence-free survival in neoadjuvant breast cancer depends on biomarker profiles: results from the I-SPY 1 TRIAL (CALGB 150007/150012; ACRIN 6657). Breast Cancer Res Treat. 2012;132(3):1049-62

© 2020 Abdel-Fatah TMA et al. *JAMA Network Open.*

eTable 8. Characteristics of Patients in the Multicenter Neoadjuvant Anthracycline-Based Combination Chemotherapy Cohort

Features		Number	Percentage
**pCR**	NO	913	85.1
	Yes	144	13.4
	Unknown	16	1.5
SPAG5	Overexpression (+)	640	59.6
	Low	433	40.4
HER2 (+) Overexpression/amplification	NO	895	83.4
	Yes	133	12.4
	Unknown	45	4.2
PR status	Negative	281	26.2
	Positive	779	72.6
	Unknown	13	1.2
Histological Grade	Low (G1)	64	6.0
	Intermediate (G2)	355	33.1
	High (G3)	230	21.4
	Unknown	424	39.5
Molecular intrinsic subtype	Basal-like	31	2.9
	Her2-enriched	28	2.6
	Luminal A	262	24.4
	Luminal B	143	13.3
	Normal	36	3.4
	Unknown	573	53.4
T Stage	T1	171	15.9
	T2	430	40.1
	T3	226	21.1
	T4	92	8.6
	Unknown	154	14.4
Pre-operative chemotherapy regimen			
Anthracycline based combination	AC	182	16.9
	FEC	86	8.0
Anthracycline based combination + Taxane	AC-T	121	11.3
	FEC-T	340	31.7
	T-FEC	293	27.3
Anthracycline based combination + Taxane + Trastuzumab	T-FEC-Trastuzumab	51	4.8

© 2020 Abdel-Fatah TMA et al. *JAMA Network Open.*

Nottingham University Hospital locally advanced breast cancer (NUH-LABC) cohort (n=361):

The relationship between SPAG5-protein expression and response to chemotherapy was evaluated by investigating its expression in pair-matched pre-chemotherapy core biopsies and post-chemotherapy surgical specimens, from 361 female patients with locally-advanced ER+ primary BC (LAP-BC) (stage IIIA-C) that had been treated with Neo-Adjuvant Anthracycline-based Combination Chemotherapy (Neo-Adj-AC-CT) at NUH between 1996 and 2015. All LAP-BC patients were female and their mean age was 50 years (range 25-75 years). Twenty-nine percent of patients received six cycles of an anthracycline-based therapy (FEC: 5-fluorouracil (5-FU) 500 mg m−2, epirubicin 75–100 mg m−2, cyclophosphamide 500 mg m−2, on day 1 of a 21 day cycle), whereas 58% of patients received FEC plus Taxane. All patients underwent mastectomy or breast-conserving surgery and axillary dissection, followed by adjuvant radiation therapy. Patients with ER+ BCs were offered 5 years of adjuvant endocrine therapy. The median follow-up time was 67 months (IRQ 27-

eTable 9. Characteristics of Patients in the Nottingham University Hospital Locally Advanced Breast Cancer Cohort

Features		Number	Percentage
SPAG5 pre-chemotherapy diagnostic core biopsy	Overexpression (+)	120	33.3
	Low (-)	221	61.2
	Unknown	20	5.5
SPAG5 post- chemotherapy surgical specimen	Overexpression (+)	39	10.8
	Low (-)	203	56.2
	pCR	43	11.9
	Unknown	76	20.1
pCR	Yes	43	72.9
	No	313	21.3
	Unknown	5	1.4
HER2 (+) Overexpression/amplification	NO	255	70.6
	Yes	104	28.8
	Unknown	2	0.6
Pre-Chemotherapy Lymph node Metastases	Negative	90	24.9
	Positive	246	68.1
	unknown	25	6.9
PR status	Negative	94	26.0
	Positive	259	71.7
	Unknown	8	2.2
Histological Grade	Low (G1)	14	3.9
	Intermediate (G2)	238	65.9
	High (G3)	100	27.7
	Unknown	9	2.5
Histological type	IDC	279	77.3
	ILC	50	13.9
	Others	26	7.2
	Unknown	6	1.7
c-TNM	IIA-IIB	63	17.5
	IIIA-IIIC	293	81.2

© 2020 Abdel-Fatah TMA et al. *JAMA Network Open.*

	Unknown	5	1.4
Tumor size (cm)	≤5	193	53.5
	>5	160	44.3
	Unknown	8	2.2
Tumor (T) Stage	T1	22	6.2
	T2	112	31.4
	T3	72	19.9
	T4	151	41.8
	Unknown	4	1.1
Pre-operative chemotherapy regimen			
Anthracycline based combination	AC/FEC	106	29.4
Anthracycline based combination + Taxane	FEC-T	208	57.6
Anthracycline based combination + Taxane + Trastuzumab	T-FEC-Trastuzumab	45	12.4
Unknown	Unknown	2	0.6
Adjuvant systemic therapy regimen	No systemic therapy	24	6.7
	Endocrine therapy alone	183	50.7
	Endocrine therapy + chemotherapy	151	41.8
	unknown	3	0.8
Adjuvant Endocrine therapy (n=334)	Tamoxifen	179	53.6
	Aromatase Inhibitors	118	35.3
	Tamoxifen+ Aromatase inhibitor	37	11.1
Trastuzumab	Yes	94	26.0
	No	265	73.4
	Unknown	2	0.6
Distant relapse	No	261	72.3
	Yes	98	27.1
	Unknown	2	0.6

81).

© 2020 Abdel-Fatah TMA et al. *JAMA Network Open.*

eTable 10. Clinicopathological Characteristics in the MD Anderson Cancer Center Cohort

Features		Number	Percentage
SPAG5 expression	Overexpression	122	40.8
	Low	177	59.2
pCR	No	257	86.0
	Yes	32	10.7
	unknown	10	3.3
HER2 (+) Overexpression/amplification	No	297	99.3
	Yes	2	0.7
PR	Negative	73	24.4
	Positive	223	74.6
	Unknown	3	1.0
Histological Grade	Low (G1)	30	10.0
	Intermediate (G2)	152	50.9
	High (G3)	100	33.4
	Unknown	17	5.7
Molecular PAM-50 intrinsic subtype	Basal-like	56	3.7
	Her2-enriched	111	7.4
	Luminal A	694	46.3
	Luminal B	471	31.4
	Normal	160	10.7
	Unknown	6	0.4
Tumor stage (T stage)	T1	34	11.4
	T2	149	49.8
	T3	68	22.7
	T4	40	13.4
	unknown	8	2.7
Lymph node Metastases	Negative	106	35.5
	Positive	190	63..5
	Unknown	3	1.0
AJCC stage	IIA	67	22.4
	IIB	83	27.8
	IIIA	75	25.1
	IIIB	57	19.1
	IIIC	17	5.7
Adjuvant systemic therapy regimen	No systemic therapy	0	0
	Endocrine therapy alone	299	100.0
	Endocrine therapy + chemotherapy	0	0
Relapse	No	257	86.0
	Yes	42	14.0

© 2020 Abdel-Fatah TMA et al. *JAMA Network Open.*

eTable 11. Characteristics of Patients in the Multicenter Adjuvant Therapy Cohort

Features		Number	Percentage
SPAG5	Overexpression (+)	640	59.6
	Low	433	40.4
HER2 (+) Overexpression/amplification	NO	737	29.2
	Yes	199	7.9
	Unknown	1585	62.9
Lymph node Metastases	Negative	1140	45.2
	Positive	942	37.4
	unknown	439	17.4
PR status	Negative	295	11.7
	Positive	1396	55.4
	Unknown	828	32.8
Histological Grade	Low (G1)	64	6.0
	Intermediate (G2)	355	33.1
	High (G3)	230	21.4
	Unknown	648	25.7
Molecular intrinsic subtype	Basal-like	31	2.9
	Her2-enriched	28	2.6
	Luminal A	262	24.4
	Luminal B	143	13.3
	Normal	36	3.4
	Unknown	1882	74.7
Tumor size (cm)	<=2	769	30.5
	>2-5	773	30.7
	>5	224	8.9
	Unknown	755	29.9
Adjuvant therapy			
Adjuvant systemic therapy regimen	No systemic therapy	459	18.2
	Endocrine therapy alone	879	34.9
	Endocrine therapy + chemotherapy	529	21.0
	unknown	654	25.9
Adjuvant Endocrine therapy (n=1408)	Tamoxifen	1376	97.7
	Aromatase Inhibitors	32	2.3
Chemotherapy (n=529)	Anthracycline based only	115	21.7
	Anthracycline based+ Taxane	348	65.8
	CMF	66	12.5
Trastuzumab	Yes	1768	70.1
	No	79	3.1
	Unknown	674	26.7
Distant relapse	No	1830	72.6

© 2020 Abdel-Fatah TMA et al. *JAMA Network Open.*

	Yes	636	25.2
	Unknown	55	2.2
Age at diagnosis (years)	<50	452	17.9
	>50	1206	47.8
	unknown	863	34.2

© 2020 Abdel-Fatah TMA et al. *JAMA Network Open.*

eTable 12. Gene Expression Platforms of Multicenter Adjuvant Therapy Cohort

Reference	Access Number	Data	Chip	Platform	Number of patients	percentage
1	GSE12093	Affymetrix	HG-U133A	GPL96	130	5.2
2	GSE2034	Affymetrix	HG-U133A	GPL96	209	8.3
3	GSE25066	Affymetrix	HG-U133A	GPL96	297	11.8
4	GSE45255	Affymetrix	HG-U133A	GPL96	89	3.5
5	GSE7390	Affymetrix	HG-U133A	GPL96	134	5.3
6	GSE16391	Affymetrix	HG-U133_Plus_2	GPL570	55	2.2
7	GSE19615	Affymetrix	HG-U133_Plus_2	GPL570	70	2.8
8	GSE21653	Affymetrix	HG-U133_Plus_2	GPL570	150	6.0
9	GSE31448	Affymetrix	HG-U133_Plus_2	GPL570	187	7.4
10	GSE42568	Affymetrix	HG-U133_Plus_2	GPL570	67	2.7
11	GSE65095	Affymetrix	HG-UA219	GPL13667	98	3.9
12-13	GSE6532-GSE9195	Affymetrix	HG-UA133A-133B, HG-UA133-PLUS2	GPL570,GPL96-GPL97	426	16.9
14	GSE22133	SWEGENE	H_v2.1.1 55K	GPL5345	227	9.0
15	GSE9893	Human Qiagen	MLRG Human 21K V	GPL5049	155	6.1
16	GSE22219	ILLUMINA	Expression BeadC	GPL6098	134	5.3
17	GSE10510	DKFZ	OLIG0-ARRAY-35K	GPL6486	93	3.7

1. Filipits M, Rudas M, Jakesz R, et al. A new molecular predictor of distant recurrence in ER-positive, HER2-negative breast cancer adds independent information to conventional clinical risk factors. Clin Cancer Res. 2011; 17(18):6012-20
2. Wang Y, Klijn JG, Zhang Y, et al. Gene-expression profiles to predict distant metastasis of lymph-node-negative primary breast cancer. Lancet. 2005;365(9460):671-9.
3. Hatzis C Pusztai L, Valero, V, et al. A genomic predictor of response and survival following taxane-anthracycline chemotherapy for invasive breast cancer. JAMA. 2011; 305(18):1873-81.
4. Nagalla S, Chou JW, Willingham MC, et al. Interactions between immunity, proliferation and molecular subtype in breast cancer prognosis. Genome Biol 2013 ;14(4):R34
5. Desmedt C, Piette F, Loi S, et al. Strong time dependence of the 76-gene prognostic signature for node-negative breast cancer patients in the

© 2020 Abdel-Fatah TMA et al. *JAMA Network Open.*

TRANSBIG multicenter independent validation series. Clin Cancer Res 2007;13(11):3207-14.
6. Desmedt C, Giobbie-Hurder A, Neven P, et al. The Gene expression Grade Index: a potential predictor of relapse for endocrine-treated breast cancer patients in the BIG 1-98 trial. BMC Med Genomics 2009 Jul 22:40.
7. Li Y, Zou L, Li Q, Haibe-Kains B, et al. Amplification of LAPTM4B and YWHAZ contributes to chemotherapy resistance and recurrence of breast cancer. Nat Med 2010 (2):214-8.
8. Sabatier R, Finetti P, Cervera N, et al. A gene expression signature identifies two prognostic subgroups of basal breast cancer. Breast Cancer Res Treat 2011;126(2):407-20.
9. Sabatier R, Finetti P, Adelaide J, et al. Down-regulation of ECRG4, a candidate tumor suppressor gene, in human breast cancer. PLoS One 2011;6(11):e27656.
10. Clarke C, Madden SF, Doolan P, et al. Correlating transcriptional networks to breast cancer survival: a large-scale coexpression analysis. Carcinogenesis 2013;34(10):2300-8.
11. Sonnenblick A, Brohée S, Fumagalli D, et al. Integrative proteomic and gene expression analysis identify potential biomarkers for adjuvant trastuzumab resistance: analysis from the Fin-her phase III randomized trial. Oncotarget 2015 ;6(30):30306-16
12. Loi S, Haibe-Kains B, Majjaj S, et al. PIK3CA mutations associated with gene signature of low mTORC1 signaling and better outcomes in estrogen receptor-positive breast cancer. Proc Natl Acad Sci U S A 2010;107(22):10208-13
13. Loi S, Haibe-Kains B, Desmedt C, et al. Predicting prognosis using molecular profiling in estrogen receptor-positive breast cancer treated with tamoxifen. BMC Genomics 2008;9:239.
14. Holm K, Staaf J, Jönsson G, et al. Characterisation of amplification patterns and target genes at chromosome 11q13 in CCND1-amplified sporadic and familial breast tumours. Breast Cancer Res Treat 2012;133(2):583-94
15. Chanrion M, Negre V, Fontaine H, et al. A gene expression signature that can predict the recurrence of tamoxifen-treated primary breast cancer. Clin Cancer Res 2008;14(6):1744-52
16. Buffa FM, Camps C, Winchester L, et al. microRNA-associated progression pathways and potential therapeutic targets identified by integrated mRNA and microRNA expression profiling in breast cancer. Cancer Res 2011;71(17):5635-45.
17. Calabrò A, Beissbarth T, Kuner R, et al. Effects of infiltrating lymphocytes and estrogen receptor on gene expression and prognosis in breast cancer. Breast Cancer Res Treat 2009;116(1):69-77.

© 2020 Abdel-Fatah TMA et al. *JAMA Network Open.*

Gene expression

SPAG5 expression profile had been established for these cancers pools using different type of microarrays including Affymetrix human microarrays [(Affymetrix®, Santa Clara, CA, USA); n= 2784 cases: MC-AT (n=1912) and MC-NACT (=872)] and Illumina [(illumina inc., San Diego, CA, USA; n=1752: METABRIC: n=1498; MC-AT (n=134) and MC-NACT (=120)]. The data from each platform were independently normalized and mapped to Ensembl gene identifiers. Affymetrix data were summarized with Ensembl alternative custom chip definition file, normalized with robust multi-array analysis, integrated with use of ComBat to remove data set –specific bias. Illumina probe profiles were quantile normalized using the lumi-package, mapped to Ensembl gene sequences using reMOAT, BioMart, and a custom BLAST sequence search. Two Agilent microarray data were resented in this study: I-SPY1 trial (accession number GSE22226) and TCGA-BRCA (obtained from FIREHOSE Broad GDAC at http://gdac.broadinstitute.org) datasets. The I-SPY1 trial were assayed on catalog 44,000 features using Agilent Human oligonucleotide microarrays (Agilent-014850 platform GPL1708) whereas TCGA-BRCA datasets were generated using RNA-seq platform (Agilent custom 244K whole genome microarrays). For SWEGENE cohort, global gene-expression analysis of BC was performed by using oligonucleotide microarrays (H_v2.1.1 55K platform GPL5345) produced at the SCIBLU Genomics Centre at Lund University, Sweden as previous described [7]. Human Qiagen microarray was used in one dataset (accession number: GSE9893; n= 155) in which gene expression profiling was performed using 70-mer oligonucleotide microarrays (22,680 oligonucleotide probes representing 21,329 human specific genes (Oligo Set 2.1 from Qiagen-Operon) as previously described. In one dataset (accession number: GSE10510; n=93) a customized microarray was constructed using DKFZ OLIG0-ARRAY-35K platform GPL6486 as previously described.

Gene expression data of each database were converted to a common scale (median equal to 0 and standard deviation equal to 1) in order to merge all of the study data that used the same platform and to create combined cohorts. Then the data was median-centred whereby median is 0. Databases using same platform have been merged and the median expression was calculated. The median expression of SPAG5 transcript for each platform has been calculated and values equal to or higher than the median coded as +1 (overexpression). Values of less than the median have been coded 0 or low SPAG5.

© 2020 Abdel-Fatah TMA et al. *JAMA Network Open.*

IHC and tissue microarray

Immunohistochemistry (IHC) of SPAG5 protein expression in breast cancer was investigated by IHC analysis in three cohorts at two independent centres: Nottingham University Hospitals (NUH), UK and University of Queensland, Australia. Immunohistochemical staining was performed using anti-SPAG5 antibody (Sigma HPA022479), at a dilution of 1:50 for 30 minutes. Negative and positive (by omission of the primary antibody and IgG-matched serum) controls were included in each run.

Nottingham University Hospital Early Stage Breast cancer (NUH-ESBC)

Tumours were arrayed in tissue microarrays (TMAs) constructed with two duplicate of 0.6mm cores from the periphery of the tumours. The TMAs were immunohistochemically profiled for SPAG5. Immunohistochemical staining was performed using the Thermo Scientific Shandon Sequenza chamber system (REF: 72110017), in combination with the Novolink Max Polymer Detection System (RE7280-K: 1250 tests), and the Leica Bond Primary Antibody Diluent (AR9352), each used according to the manufacturer's instructions (Leica Microsystems). The tissue slides were deparaffinised with xylene and then rehydrated through five decreasing concentrations of alcohol; 100%, 90%, 70%, 50% and 30% for two minutes each. Pre-treatment antigen retrieval was performed on the TMA sections using sodium citrate buffer (pH 6.0) and heated for 20 minutes at 95˚C in a microwave (Whirpool JT359 Jet Chef 1000W). A set of slides were incubated with the primary anti-SPAG5 antibody (Sigma HPA022479), at a dilution of 1:50 for 30 minutes. Negative and positive (by omission of the primary antibody and IgG-matched serum) controls were included in each run. The negative control ensured that all the staining was produced from the specific interaction between antibody and antigen.

The individual tissue cores were scored by two experienced pathologists, according to the intensity of tumour cell staining: punctate cytoplasmic staining was recorded as 1+ (if it is of mild/moderate granular intensity) or 2+ (if it is with strong granular intensity). No staining, faint or diffuse staining was recorded as 0 (negative or week). Using the maximum score of duplicate tissue cores for each case, the percentage of each category was estimated (0-100%). A score (range 0-200) was calculated by multiplying intensity of staining and percentage staining. A median score of ≥ 1 was taken as the cut-off for high SPAG5 cytoplasmic expression. Not all cores within the TMA were suitable for IHC analysis as some cores were missing or lacked tumour (<15% tumour). The expression of HER2, ER and PR was re-assessed according to the American Society of Clinical Oncology/College of American Pathologists (ASCO/CAP) guidelines. To validate the use of TMAs for immuno-phenotyping, full-face sections of 40 cases were stained and the protein expression levels were compared. The concordance between TMAs and full-face sections was excellent using Cohen's kappa statistical test for categorical variables (kappa=0.8).

Nottingham University Hospital Locally Advanced Breast cancer (NUH-LABC)

Full face sections from the diagnostic pre-chemotherapy core biopsies (n=361) and post-chemotherapy residual tumours in the surgically removed specimens were stained with **SPAG5 as mentioned above.**

Queensland breast cancers follow up (QLBCFU)

Four u TMA sections were processed in a decloaker for antigen retrieval in sodium citrate buffer (pH 6.0) for 20 mins, and then IHC was performed using an anti-SPAG5 antibody (Sigma HPA022479; 1:50), and the Mach 1 Universal HRP-Polymer Detection kit (Biocare Medical). Haematoxylin-counterstained, mounted sections were then scanned at 40 x magnification on an Aperio AT Turbo slide scanner (Leica Biosystems). Digital images of individual tissue cores were scored by three experienced molecular pathologists according to the intensity of tumour cell staining: punctate cytoplasmic staining was recorded as 1+ (moderate) or 2+ (strong), and multiplied by the percentage of tumour cells stained to derive a final score ranged (0 to 200). Faint, diffuse staining was recorded as 0. Using the maximum score of duplicate tissue cores for each case, associations between SPAG5 expression and clinicopathological variables were investigated.

1. Wolff AC, Hammond ME, Schwartz JN, et al. American Society of Clinical Oncology/College of American Pathologists guideline recommendations for human epidermal growth factor receptor 2 testing in breast cancer. J Clin Oncol 2007; 25: 118-45.
2. Hammond ME, Hayes DF, Wolff AC, Mangu PB, Temin S. American society of clinical oncology/college of american pathologists guideline recommendations for immunohistochemical testing of estrogen and progesterone receptors in breast cancer. J Oncolo Pract 2010; 6: 195-7.

© 2020 Abdel-Fatah TMA et al. *JAMA Network Open.*

eTable 13. Association of SPAG5 mRNA Expression and Clinicopathologic Variables in the Molecular Taxonomy of Breast Cancer International Consortium Cohort

	Low<br>N (%)	High<br>N (%)	X2<br><br>Adjusted p value
**Lymph node (LN) metastases**			
Negative	467 (55.6%)	349 (53.0%)	0.324
Positive	373 (44.4%)	309 (47.0%)	
**Grade****			<0.0001*
Low (G1)	118 (14.9%)	40 (6.3%)	
Intermediate (G2)	431 (54.3%)	277 (43.3%)	
High (G3)	245 (30.9%)	323 (50.5%)	
**Tumour Size (cm)**			0.051
T 1a+b(1·0)	47 (5.6%)	20 (3.1%)	
T 1c(>1·0-2·0)	242 (29.0%)	176 (27.1%)	
T2 (>2·0-5)	509 (61.0%)	430 (66.2%)	
T3 (>5)	37 (4.4%)	24 (3.7%)	
**PR (IHC)**<br>Negative	254 (30.2%)	261 (39.7%)	<0.0001*
Positive	586 (69.8%)	397 (60.3%)	
Her2 overexpression<br>No<br>Yes	 811 (96.5%)<br>29 (3.5%)	 572 (86.9%)<br>86 (13.1%)	<0.0001*
**HER2 gene status using SNP 6 (Chip)**			
Loss	50 (6.0%)	25 (3.8%)	<0.0001*
Neutral	697 (83.3%)	444 (67.8%)	
Gain	90 (10.8%)	186 (28.4%)	
**TP53 mutation status**			
Wilde type	343 (94.2%)	250 (88.7%)	0.01*
Mutant	21 (5.8%)	32 (11.3%)	
**Ki67 transcript**<br>Low<br>High	 625 (74.4%)<br>215 (25.6%)	 232 (35.3%)<br>426 (64.7%)	<0.001*
**Genous subtype using Genfu**			<0.0001*

© 2020 Abdel-Fatah TMA et al. *JAMA Network Open.*

**software**			
ER+/Her-2 negative/low proliferation	301(66.6%)	55 (16.3%)	
ER+/Her-2 negative/high proliferation	120 (26.5%)	230 (68.0%)	
Her-2 positive	14 (3.1%)	43(12.7%)	
ER-/Her-2 negative	17(3.8%)	10 (3.0%)	
**PAM50 subtype**<br>PAM50·LumA<br>PAM50·LumB<br>PAM50·Her2<br>PAM50·Basal<br>PAM50·Normal	 524 (62.7%)<br>121 (14.5%)<br>32 (3.8%)<br>23 (2.8%)<br>136 (16.3%)	 170 (25.9%)<br>350 (53.4%)<br>79 (12.0%)<br>33 (5.0%)<br>24 (3.7%)	<0.0001*
**Integrated Clusters (IntClust)**<br>intClust·1<br>intClust·2<br>intClust·3<br>intClust·4<br>intClust·5<br>intClust·6<br>intClust·7<br>intClust·8<br>intClust·9<br>intClust·10	 <br>24 (2. 9)<br>29 (3.5%)<br>201 (23.9%)<br>203 (24.2%)<br>18 (2.1%)<br>37 (4.4%)<br>104 (12.4%)<br>180 (21.4%)<br>36 (4.3%)<br>8 (1.0%)	 <br>97 (14.7%)<br>39 (5.9%)<br>73(11.1%)<br>48 (7.3%)<br>67 (10.2%)<br>45 (6.8%)<br>73 (11.1%)<br>111 (16.9%)<br>83 (12.6%)<br>22 (3.3%)	<0.0001*

* Statistically significant at p<0·05; **: grade as defined by Nottingham grading system (NGS); ER: oestrogen receptor; PR: progesterone receptor; HR: hormone receptor; HER2: human epidermal growth factor receptor 2; Triple negative: ER-/PR-/HER2-.

© 2020 Abdel-Fatah TMA et al. *JAMA Network Open.*

eTable 14. Association of SPAG5 mRNA Expression and Clinicopathologic Variables in the Swegene Cohort

	**Low**<br>**N (%)**	**High**<br>**N (%)**	X2<br><br>Adjusted p value
**Grade****			<0.0001*
Low (G1)	21 (24.7%)	1 (1.9%)	
Intermediate (G2)	50 (58.8%)	27 (50.0%)	
High (G3)	14 (16.5%)	26 (48.1%)	
**Intrinsic**<br>**subtype**<br>LumA<br>LumB<br>Her2<br>Basal<br>Normal	 69 (50.0%)<br>18 (13.0%)<br>4 (3.0%)<br>2 (1.4%)<br>23 (16.7%)	 20 (22.5%)<br>46 (51.7%)<br>6 (6.7%)<br>3 (3.4%)<br>1 (1.1%)	<0.0001*
Unknown	22 (15.9)	13 (14.6	
**Integrated**<br>**Clusters**<br>**(IntClust)**<br>17q12<br>Amplifier<br>Basal<br>Complex<br>Luminal<br>Complex<br>Luminal<br>Simple<br>Mixed	 <br>8 (5.8)<br>25 (18)<br>1 (0.7)<br>43 (31.2)<br>42 (30.4)<br>19 (13.8)	 <br>11 (12.4)<br>15 (16.9)<br>6 (6.7)<br>48 (53.9)<br>2 (2.2)<br>7 (7.9)	<0.0001*

* Statistically significant at p<0·05; **: grade as defined by Nottingham grading system (NGS); ER: oestrogen receptor; PR: progesterone receptor; HR: hormone receptor; HER2: human epidermal growth factor receptor 2; Triple negative: ER-/PR-/HER2-.

© 2020 Abdel-Fatah TMA et al. *JAMA Network Open.*

eTable 15. Clinicopathological Association of SPAG5 Protein Expression in the Nottingham Historical Early Stage Breast Cancer Cohort

Variables	SPAG5 protein expression		X2
	Low	High	Adjusted p value
Tumour size<br>T1 a+ c (=<1·0)<br>T2 –T3(>2·0)	 569 (88·9)<br>304 (84·7)	 71 (11·1)<br>55 (15·3)	0·054
Lymph node stage<br>Negative<br>Positive	 545 (62·4)<br>329 (37·6)	 68 (53·5)<br>59 (46·5)	0·057
Tumour grade<br>G1<br>G2<br>G3	 204 (23·4)<br>386 (44·2)<br>283 (32·4)	 17 (13·5)<br>35 (27·8)<br>74 (58·7)	<0·0001*
Mitotic index<br>M1 (low; mitoses <10)<br>M2 (medium; mitoses 10-18)<br>M3 (high; mitoses >18)	 428 (49·2)<br>193 (22·2)<br>249 (28·6)	 32 (25·6)<br>24 (19·2)<br>69 (55·2)	<0·0001*
Progesterone receptor<br>Negative<br>Positive	 177 (21·4)<br>651 (64·9)	 21 (17·2)<br>101 (82·8)	0.290
Androgen receptor<br>Negative<br>Positive	 167 (23.5)<br>545 (76.5)	 38 (35.8)<br>68 (64.2)	0·006*
EGFR<br>Negative<br>Overexpression	 608 (86·1)<br>98 (13·9)	 85 (76·6)<br>26 (23·4)	0·009*
HER2<br>Negative<br>Overexpression	 813 (94·6)<br>46 (5·4)	 106 (83·5)<br>21 (16·5)	<0·0001*
4 IHC (liminal A)	320 (44.1)<br>405 (55.9)	80 (69.6)<br>35 (30.4)	<0·0001*
4 IHC (liminal A)	457 (62.9)<br>269 (37.1)	56 (48.7)<br>59 (51.3)	0·004*
4 IHC (HER2+)	813 (94.6)<br>46 (5.4)	106 (83.5)<br>21 (16.5)	<0·0001*
p53<br>Negative<br>Positive	 656 (90·7)<br>67 (9·3)	 85 (75·9)<br>27 (24·1)	<0·0001*
MDM2<br>Negative<br>Overexpression	 434 (70·0)<br>186 (30·0)	 88 (84·6)<br>16 (15·4)	0·002*

© 2020 Abdel-Fatah TMA et al. *JAMA Network Open.*

p16<br>Negative<br>Overexpression	 591 (96·7)<br>20 (3·3)	 88 (90·7)<br>9 (9·3)	0·006*
Bcl2<br>Negative<br>Positive	 161 (20·0)<br>643 (80·0)	 34 (27·6)<br>89 (72·4)	0·054
Cyclin B2<br>Negative<br>Positive	 452 (71·6)<br>371 (28·4)	 43 (37·4)<br>72 (62·6)	<0·0001*
Cyclin E<br>Negative<br>Positive	 284 (92·2)<br>24 (7.8)	 40 (80·04)<br>72 (62·6)	0·006*
Ki67<br>Negative<br>Positive	 338 (46·2)<br>394 (58·8)	 23 (20·4)<br>90 (79·6)	<0·0001*
CDK1<br>Negative<br>Positive	 328 (78·8)<br>88 (21.2)	 49 (68·1)<br>23 (31·9)	0·04*
BRCA1<br>Negative<br>Positive	 80 (12·6)<br>555(87·4)	 23 (22·8)<br>78 (77·2)	0·006*
SRC3	 431 (57·9)<br>314(42·1)	 50 (45·5)<br>60 (54·5)	0·014*
Phospho-SRC3-T24	 462 (72·4)<br>176(27·6)	 81 (85·3)<br>14 (14·7)	0·008*

© 2020 Abdel-Fatah TMA et al. *JAMA Network Open.*

eTable 16. Multivariable Cox Regression Models Analysis for 5-Year Overall Survival in the Nottingham University Hospital Early Stage Breast Cancer Cohort

Variables	HR	95.0% CI		P value
		Lower	Upper	
**SPAG5 (+)**	1.68	1.18	2.39	**0.004***
**PR (+)**	0.54	0.40	0.73	**<0.0001***
**HER2 (+)**	1.68	1.22	2.32	**0.002***
**Tumour Size (continuous)**	1.59	1.23	2.07	**<0.0001***
**Lymph node (LN) status (+)**	1.72	1.44	2.05	**<0.0001***
**Histological grade (G3 vsG2-G1)**	2.63	2.10	3.30	**<0.001***
**Hormone therapy**	0.08	0.76	0.56	0.079
**Chemotherapy**	0.68	0.48	0.94	**0.022***

*Statistically significant at p<0.05

© 2020 Abdel-Fatah TMA et al. *JAMA Network Open.*

eTable 17. Multivariable Cox Regression Models Analysis for 5-Year Overall Survival in Queensland Breast Cancer Follow-Up Cohort

Parameters	Univariate analysis (Log-rank or Gehan-Breslow Wilcoxon*)				Multivariate analysis (Stepwise cox proportional-hazards)		
	No death/ no survivor	Rate	Differential	p	HR	95% CI	p
Lymph Node Status				<0.0001			0.0001
Negative	100/133	0.25					
Positive	40/108	0.63	2.54		2.69	1.63 - 4.46	
HER2 Status				<0.0001			
Negative	291/445	0.35					
Overexpression	17/57	0.70	2.03		2.73	1.51 - 4.93	0.0009
Histological Grade				<0.0001			
G1	60/74	0.19					NS
G2	152/249	0.39	2.06				
G3	97/171	0.43					
Lymphovascular Invasion				<0.0001			NS
Absent	274/413	0.34					
Present	51/109	0.53	1.58				
SPAG5 Expression				<0.0001			
Low	262/404	0.35					
Overexpression	34/73	0.53	1.52		1.92	1.11 - 3.35	0.021
Tumour Size							
<= 1.5 cm	42/156	0.27		0.0005	2.4	1.25-4.6	0.009
> 1.5	90/208	0.43	1.29				
Ki67 expression				0.0005			NS
Low	259/409	0.37					
High	33/64	0.48	1.32				

Gehan-Breslow-Wilcoxon p values are shown for parameters with most prognostic significance within the first 5 years post-

© 2020 Abdel-Fatah TMA et al. *JAMA Network Open.*

eFigure 1. Clinical Outcome of *SPAG5* Copy Number Variants and Transcript Expression and SPAG5 Protein Expression in the Estrogen Receptor–Positive Breast Cancer

All cases METABRIC (n=1498)

SPAG5 transcript

A

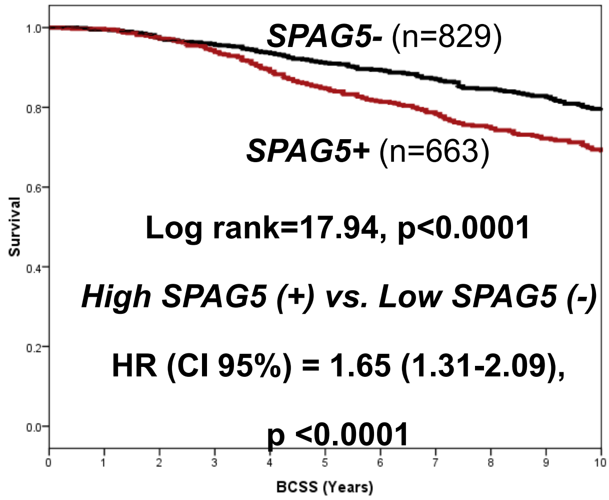

B

ER+NUH-ESBC (n=1970)

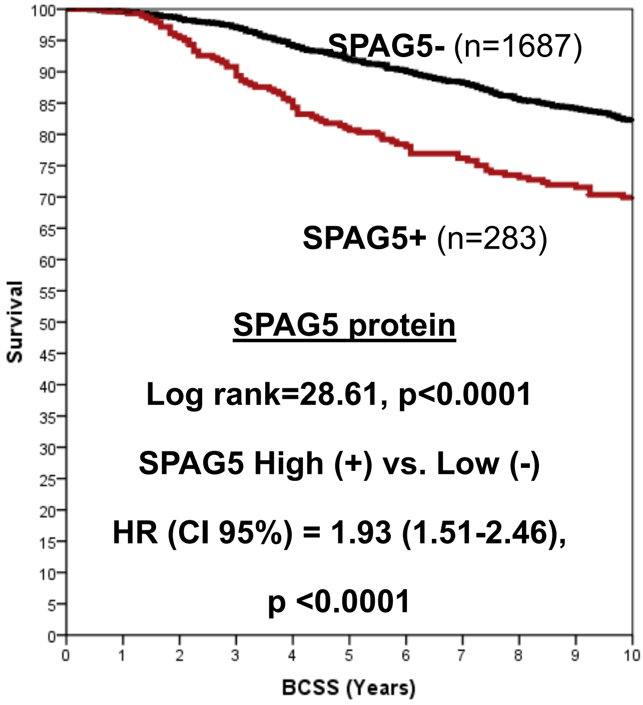

C

ER+ QFU cohort (n= 287)

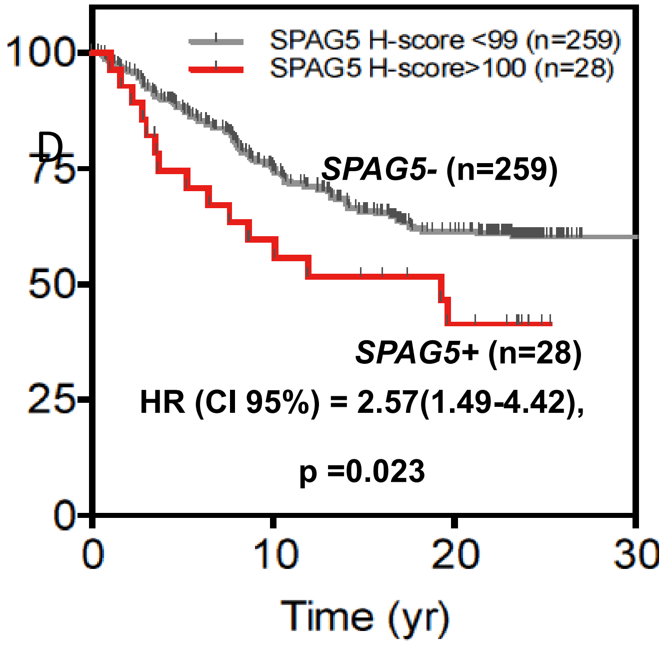

© 2020 Abdel-Fatah TMA et al. *JAMA Network Open.*

eFigure 2. Clinical Outcome of *SPAG5* Transcript and SPAG5 Protein Expression in the Molecular Taxonomy of Breast Cancer International Consortium and Nottingham University Hospital Early Stage Breast Cancer Cohorts

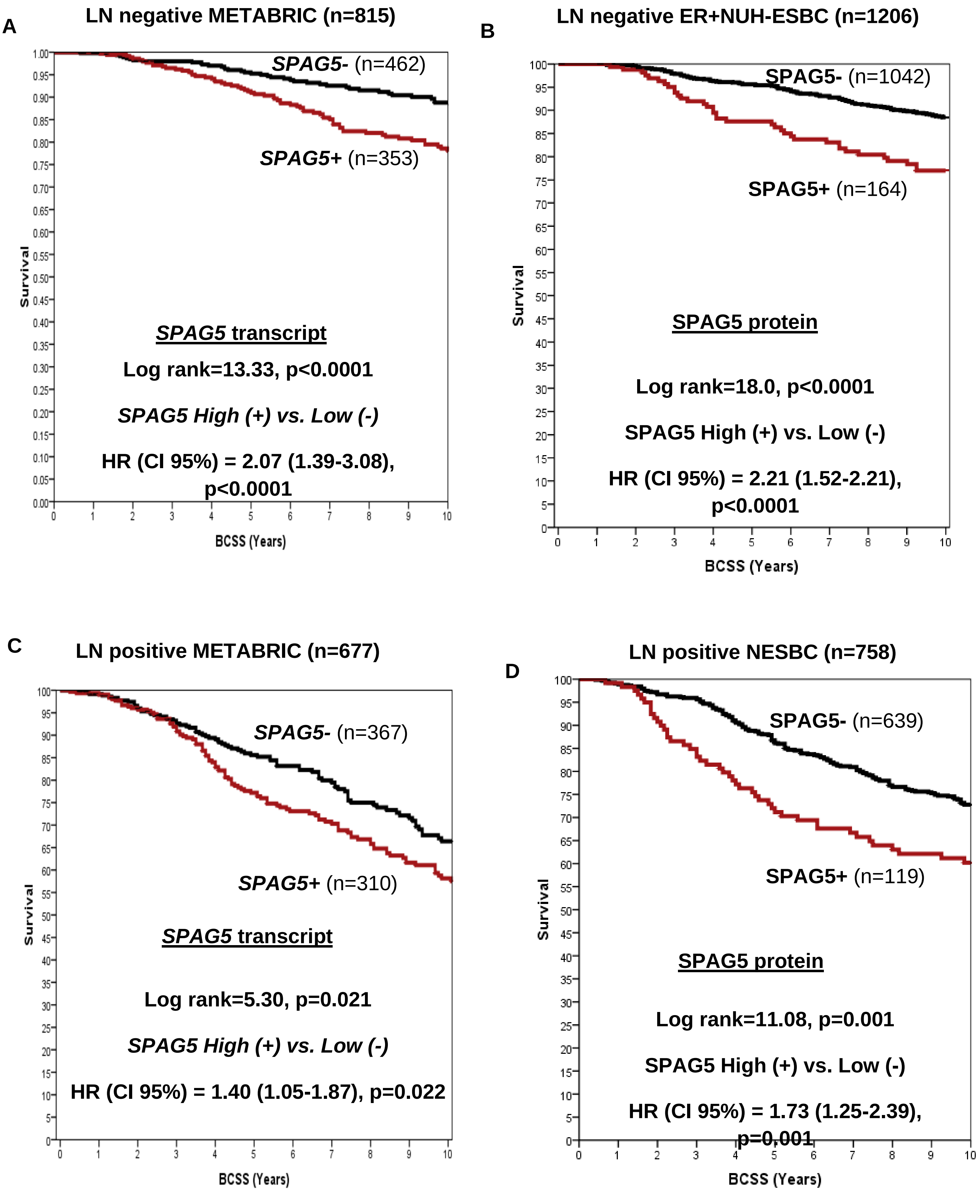

© 2020 Abdel-Fatah TMA et al. *JAMA Network Open.*

eFigure 3. Clinical Outcome of *SPAG5* Amplification Transcriptomic Signature

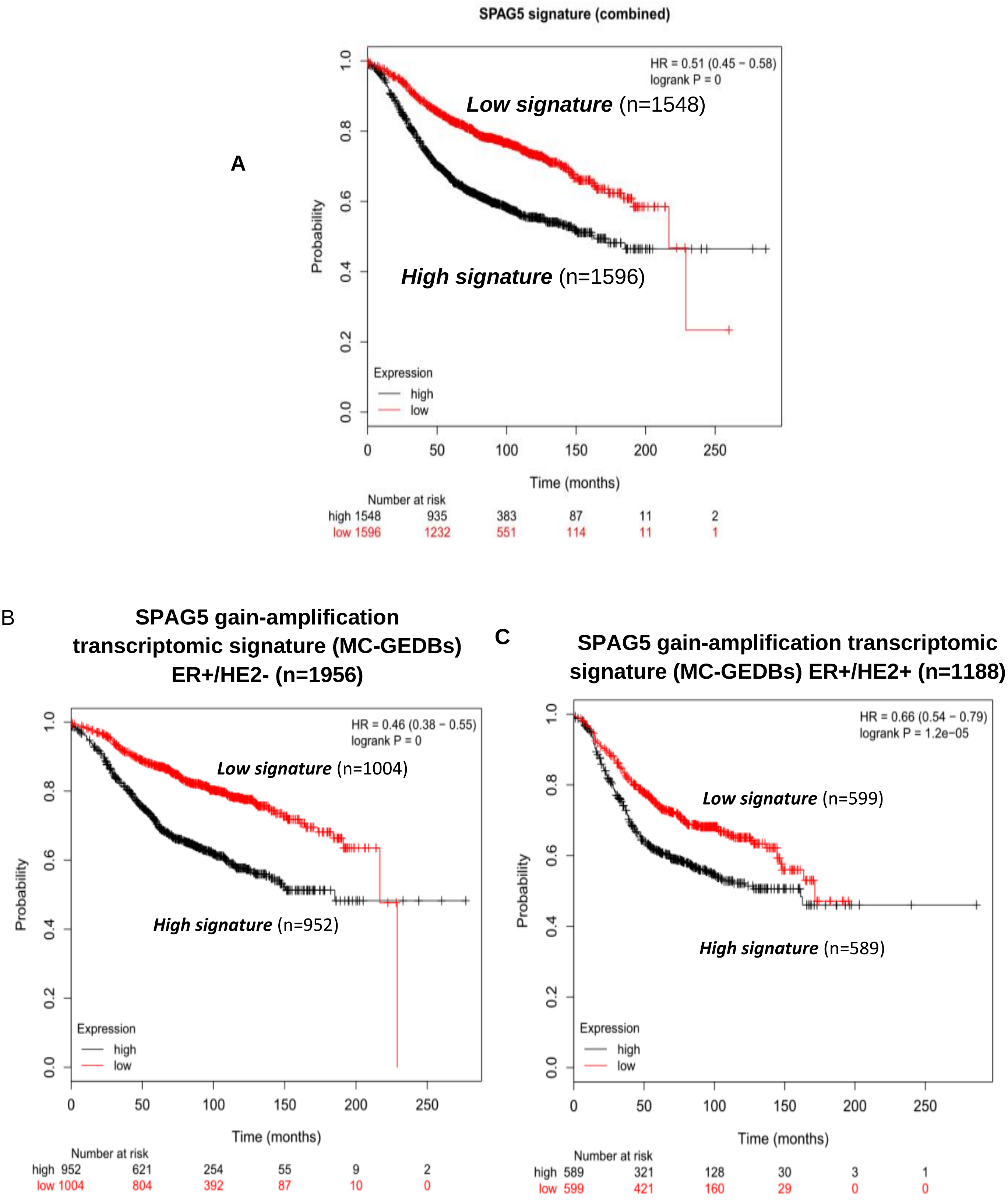

© 2020 Abdel-Fatah TMA et al. *JAMA Network Open.*

eFigure 4. *SPAG5* Transcript Expression and Clinical Response to Neoadjuvant Endocrine Therapy

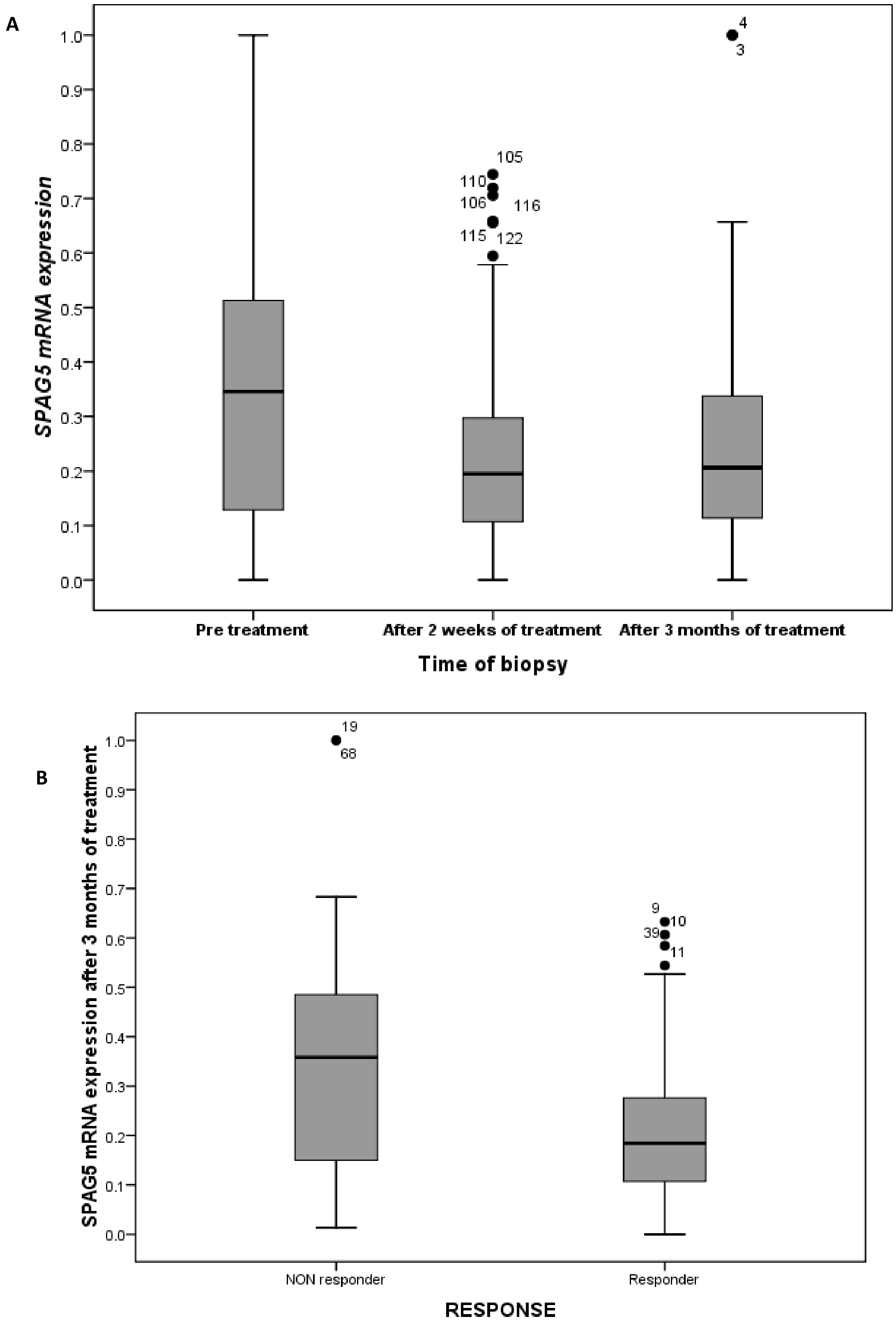

© 2020 Abdel-Fatah TMA et al. *JAMA Network Open.*

eFigure 5. *SPAG5* Transcript Expression and Clinical Response to Neoadjuvant Endocrine Therapy

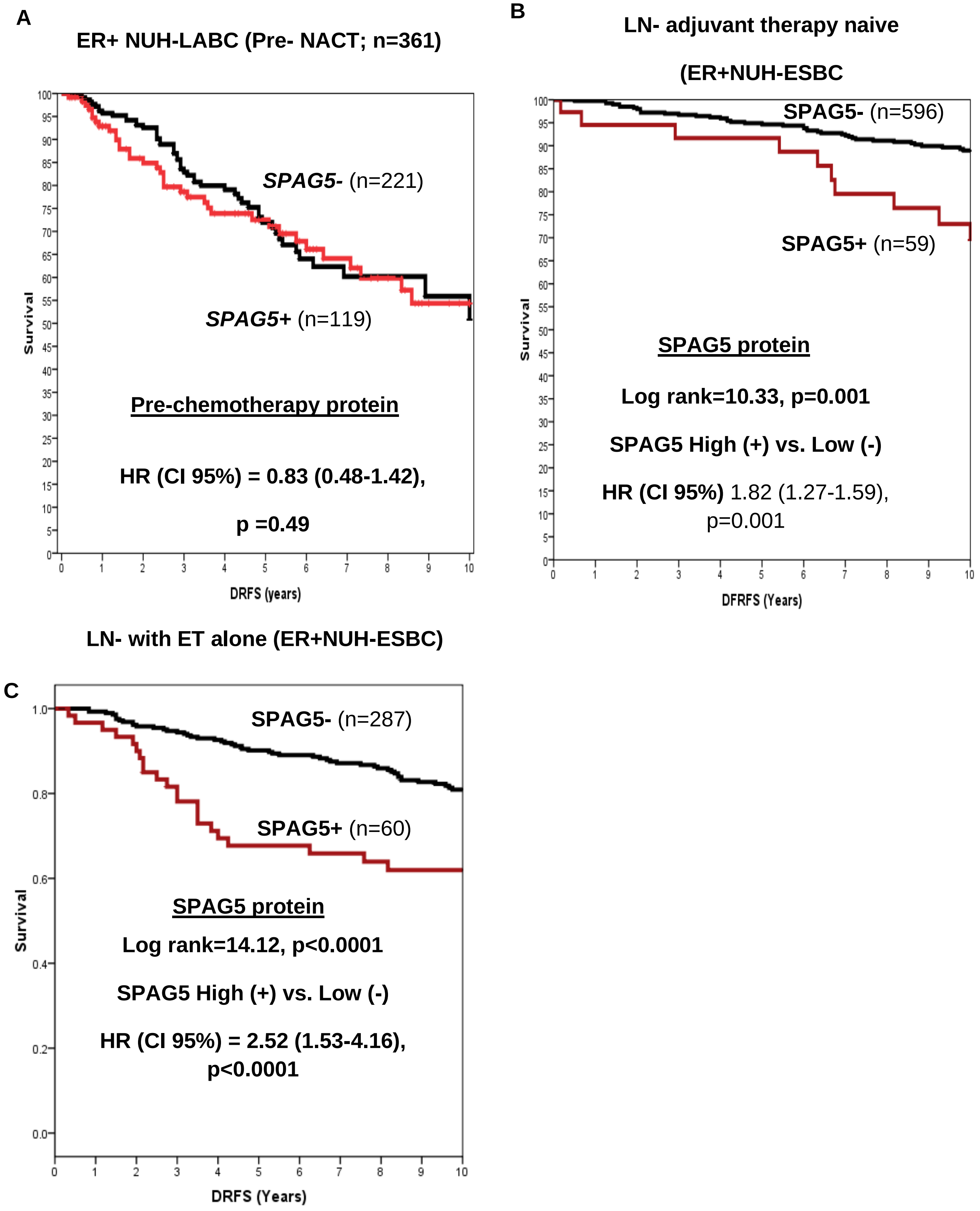

© 2020 Abdel-Fatah TMA et al. *JAMA Network Open.*

eFigure 6. Kaplan-Meier Curves Showing the Outcomes of the Received Adjuvant Systemic Therapy on Distant Relapse Free Survival in Patients With Low and High *SPAG5* Transcript, Without Lymph Node Involvement and High or Low *SPAG5* Transcript in the Multicenter Adjuvant Therapy Cohort

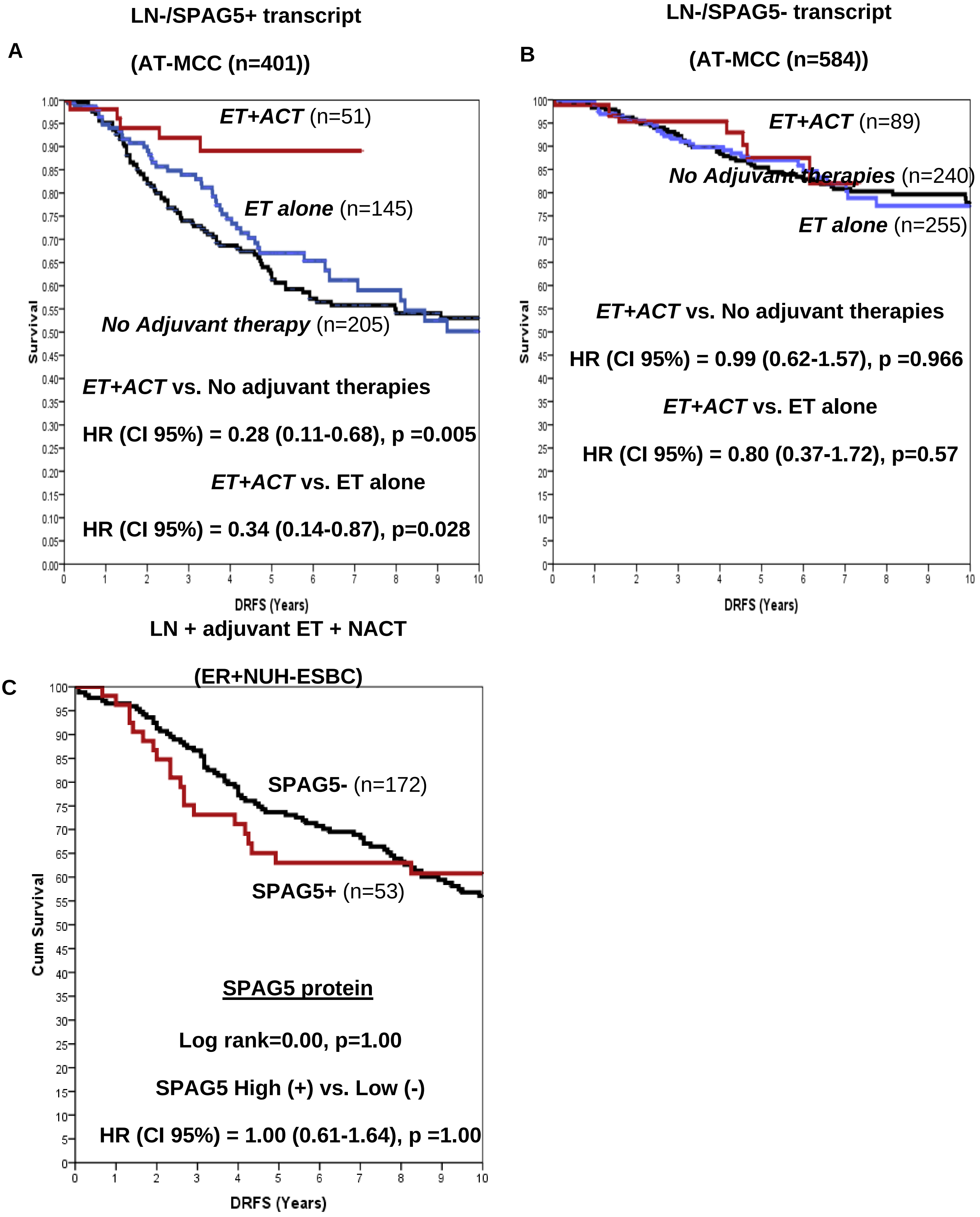

© 2020 Abdel-Fatah TMA et al. *JAMA Network Open.*
